# Heart rate variability during head‐up tilt shows inter‐individual differences among healthy individuals of extreme *Prakriti* types

**DOI:** 10.14814/phy2.15435

**Published:** 2022-09-15

**Authors:** Ritu Rani, Prathiban Rengarajan, Tavpritesh Sethi, Bharat Krushna Khuntia, Arvind Kumar, Deep Shikha Punera, Deepika Singh, Bhushan Girase, Ankita Shrivastava, Sanjay K. Juvekar, Bala Pesala, Mitali Mukerji, Kishore Kumar Deepak, Bhavana Prasher

**Affiliations:** ^1^ Centre of Excellence for Applied Development of Ayurveda Prakriti and Genomics CSIR‐Institute of Genomics & Integrative Biology Delhi India; ^2^ CSIR's Ayurgenomics Unit–TRISUTRA (Translational Research and Innovative Science ThRough Ayurgenomics) CSIR‐Institute of Genomics and Integrative Biology New Delhi India; ^3^ Genomics and Molecular Medicine CSIR‐Institute of Genomics & Integrative Biology Delhi India; ^4^ Academy of Scientific and Innovative Research Ghaziabad Uttar Pradesh India; ^5^ Department of Physiology All India Institute of Medical Sciences New Delhi India; ^6^ Vadu Rural Health Program KEM Hospital Research Centre Pune India; ^7^ Indraprastha Institute of Information Technology Delhi India; ^8^ Indian Institute of Technology Jodhpur Rajasthan India

**Keywords:** autonomic nervous system, Ayurgenomics, Ayurveda, head‐up tilt, heart rate variability, orthostatic stress, *Prakriti*

## Abstract

Autonomic modulation is critical during various physiological activities, including orthostatic stimuli and primarily evaluated by heart rate variability (HRV). Orthostatic stress affects people differently suggesting the possibility of identification of predisposed groups to autonomic dysfunction‐related disorders in a healthy state. One way to understand this kind of variability is by using *Ayurvedic* approach that classifies healthy individuals into *Prakriti* types based on clinical phenotypes. To this end, we explored the differential response to orthostatic stress in different *Prakriti* types using HRV. HRV was measured in 379 subjects(*Vata* = 97, *Pitta* = 68, *Kapha* = 68, and Mixed *Prakriti* = 146) from two geographical regions(Vadu and Delhi NCR) for 5 min supine (baseline), 3 min head‐up‐tilt (HUT) at 60°, and 5 min resupine. We observed that *Kapha* group had lower baseline HRV than other two groups, although not statistically significant. The relative change (%*Δ*_1&2_) in various HRV parameters in response to HUT was although minimal in *Kapha* group. *Kapha* also had significantly lower change in HR, LF (nu), HF (nu), and LF/HF than *Pitta* in response to HUT. The relative change (%*Δ*_1_) in HR and parasympathetic parameters (RMSSD, HF, SD1) was significantly greater in the *Vata* than in the *Kapha*. Thus, the low baseline and lower response to HUT in *Kapha* and the maximum drop in parasympathetic activity of *Vata* may indicate a predisposition to early autonomic dysfunction and associated conditions. It emphasizes the critical role of *Prakriti*‐based phenotyping in stratifying the differential responses of cardiac autonomic modulation in various postures among healthy individuals across different populations.

## Graphical abstract

*Differential response to Orthostatic stress among Prakriti groups*: Healthy individuals stratified into Ayurveda *Prakriti* groups, subjected to orthostatic stress (head up tilt), and had heart rate (HR) and heart rate variability measured (upper panel). Lower panel a and b depict the response (*Δ*1 & 2) to orthostatic stress. Each color line represents *Prakriti* groups in the order corresponding to the magnitude of change observed. Response was seen greater in the *Vata* group in HR and parasympathetic parameters. Whereas *Kapha Prakriti* individuals were least responsive in HUT.

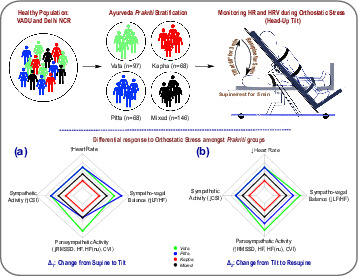

## 1 INTRODUCTION

Postural alterations are an important aspect of daily life, which have an enormous influence on our physical health and well‐being. For instance, the sudden upright posture stance causes displacement of the blood volume, which unloads the carotid and cardiopulmonary baroreceptors, causing dynamic activation of noradrenergic sympathetic outflow (Ichinose et al., 2008). As a result, peripheral vasoconstriction increases, and there is a decrease in venous return, stroke volume, and cardiac output (Zaidi, 2000). Movement is logistical support by the interplay of the autonomic nervous system and its effectors even if only taking a step or rising from lying to standing position (Watanabe et al., 2007). However, if compensatory reflex system activation is unbalanced or impaired, then individuals will experience syncope, orthostatic hypotension, and other postural intolerance issues. To evaluate these autonomic nervous system activities, heart rate variability (HRV) is used as a key parameter (Acharya et al., 2006; Malik et al., 1996; Pomeranz et al., 1985; Watanabe et al., 2007). HRV is calculated from the variation in the time interval of the successive cardiac cycles (Kumar et al., 2021; Shaffer & Ginsberg, 2017). In general, higher HRV reflects a healthy condition, whereas decreased HRV reflects a diseased condition. Studies have linked low HRV and autonomic dysfunction to conditions such as coronary artery disease, mitral valve disease, syncope, osteoporosis, arthritis, diabetes, psychological illness, and certain cancers, as well as muscle weakness, frailty, and disability (Ernst, 2017; Kristal‐Boneh et al., 1995; Thayer et al., 2010).

HRV research has revealed inter and intra‐individual variations among healthy (Lischke et al., 2019). Individual HRV variations and emotional outcomes were found to be positively correlated. People with lower HRV have a higher negative motivational value when faced with unfavorable images (Katahira et al., 2014). Inter‐individual differences in vagally mediated HRV (HF‐HRV) and empathy and alexithymia were also studied (Lischke et al., 2018). The category and dimensional analyses revealed that high HRV people had more empathy and lower alexithymia than low HRV people (Lischke et al., 2018). The Japanese study found higher inter‐individual than intra‐individual variations in healthy males at rest (Kobayashi, 2007). Resting HRV was measured in supine and standing position and the coefficient variation of HR, HF, and LF were found to vary between individuals. The log‐transformed HRV indices had inter‐individual variations of 7%–9%, while the time‐domain indices (SDNN and rMSSD) had high inter‐individual variabilities of 43%–44% (Kobayashi et al., 2012). Thus, studies suggest that HRV does reflect the physiological makeup of an individual.

To gain a better understanding of the cardiovascular regulation mechanism that occurs during such orthostatic stress, the head‐up tilt (HUT) test is extensively used. Several studies have demonstrated that even healthy individuals respond differently to HUT (Natale et al., 1995; Petersen et al., 2000). When compared to the low heart rate group, the high heart rate group had greater changes in systolic arterial pressure, pulse pressure, and norepinephrine levels during HUT test (Ramirez‐Marrero et al., 2008). Healthy adults with increased heart rate responses to tilt have similar cardiovascular characteristics to those found in patients with Postural Orthostatic Tachycardia Syndrome (POTS) (Ramirez‐Marrero et al., 2008). In general, healthy persons ‘HUT’ responses are diverse, which complicates the understanding of the pathophysiology of orthostatic diseases. However, there is a general lack of research on the variability of HUT responses. We believe that understanding HRV in terms of HUT response can help predict an individual's physiological reaction and thereby disease susceptibility and early disease prediction. We, therefore, need a phenotypic stratification framework to identify and measure variability in response among healthy populations.

In Ayurveda, the ancient Indian system of medicine, individuals are classified into constitution types “*Prakriti*” based on the assessment of multisystem phenotypes (Prasher et al., 2008, 2016; Sethi et al., 2011). According to Ayurveda, each individual is born with a specific proportion of *Tri‐dosha (Vata, Pitta, and Kapha)* which determines their basic constitution termed “*Prakriti*”. Individuals in a population (healthy as well as diseased) can be stratified into seven broad groups (*Vata, Pitta, Kapha, Vata‐Pitta, Vata‐Kapha, Pitta‐Kapha, & Vata‐Pitta‐Kapha*) with nearly 8%–10% of them consisting of extreme, contrasting *dosha Prakriti* (*Vata, Pitta, Kapha*) and distinct disease predispositions (Govindaraj et al., 2015; Prasher et al., 2008). The rest of the population consists of mixed *Prakriti*, including dual *dosha* dominant *Vata‐Pitta, Vata‐Kapha*, and *Pitta‐Kapha* as well as the three *doshas* dominant *Vata‐Pitta‐Kapha Prakriti* groups. These constitution types also differ with respect to their physiological responses and some of these attributes are examined during the assessment of *Prakriti* for example food habits and digestive capacity, taste preferences, tendency to gain and lose weight, tolerance for specific weather, and response to stress (Prasher et al., 2008, 2016; Sethi et al., 2011). Our earlier studies were able to explain inter‐individual variability at the genetic, genomic, molecular, microbiome, and cellular levels by utilizing a unique Ayurgenomics framework (Aggarwal et al., 2010, 2015; Chakraborty et al., 2021; Chauhan et al., 2018; Prasher et al., 2008). The baseline differences have also been shown to predict differences in response to hypoxia that are relevant in high altitude adaptation and thrombotic outcomes (Aggarwal et al., 2010, 2015). Earlier studies by Tripathi et al. (2011) have shown an increase in diastolic blood pressure immediately after 5‐min isotonic exercise in the dual *Prakriti* group of *Vata‐Pitta* and *Pitta‐Kapha* group, whereas this change in increase in the *Vata‐Kapha* group is minimal. Considering the significance of orthostatic stimuli in health and diseases, the current study is conducted to analyze short HRV responses before, during, and re‐supine phases of HUT in extreme constitution types to explore *Prakriti* specific differences can be observed. In this study, we observed the low baseline HRV and relative change in *Kapha* and the maximum parasympathetic withdrawal in *Vata* in response to HUT. In particular, we were able to capture inter‐individual variability through cardiac autonomic modulation (HRV) in response to HUT challenge among healthy individuals segregated on the basis of *Prakriti*.

## 2 MATERIALS AND METHODS

### 2.1 Details of cohort 1

The study was conducted at KEMHRC, Vadu cohort of the Ayurgenomics project. Vadu cohort was derived from a population of about 140,000 under health and demographic surveillance since 2002 in rural Pune district of Western India (Patil et al., 2019). And 10,100 individuals were pre‐screened with age groups 18–40 years of an equal number of healthy males and females (Tiwari et al., 2017). The health of the individual was assessed by self‐reporting. All experiments and procedures were conducted as per the guidelines provided by the committee and the protocols are approved by the Institutional Human Ethics Committee of CSIR‐IGIB and KEMHRC, Vadu. Written informed consent was obtained from all the subjects prior to induction in the study. The detailed *Prakriti* evaluation was carried out in 528 individuals for *Prakriti* stratification by trained Ayurveda clinicians using the questionnaire that has been developed at CSIR‐IGIB. Exclusion criteria included presenting any feature of systemic disorders, on any kind of medications, pregnant and lactating women or presenting any signs of hormonal imbalance. For this study, 255 individuals' baseline and HUT test HRV data are analyzed. Out of 255 subjects, 68, 36, 51, and 100 were *Vata*, *Pitta*, *Kapha*, and Mixed *Prakriti*, respectively.

### 2.2 Details of cohort 2

Totally 927 apparently healthy individuals are screened for *Prakriti* assessment. Out of these, 78 subjects are found to be extreme *Prakriti* and recruited for HUT, along with 46 subjects identified as Mixed *Prakriti* for background control. Among 78 subjects, 29, 32, and 17 are extreme *Vata, Pitta*, and *Kapha Prakriti*, respectively. The subjects were recruited from various colleges and research institutions from Delhi, NCR, after their written consent. All the experiments and protocols are approved by the Institutional Ethical Committee of AIIMS, New Delhi.

### 2.3 Heart rate variability recording procedure for both the cohorts

A total of 379 volunteers were included for the HUT test among them 97, 68, 68, were extreme *Vata*, *Pitta*, and *Kapha Prakriti* groups, respectively and 146 Mixed *Prakriti* (*Vata‐Pitta* = 82, *Kapha‐Pitta* = 51, *Vata‐Kapha* = 13) groups from both the cohorts.

Each individual was instructed to have a light breakfast at least 2 h before and allowed to relax for half an hour before the HRV recording. At the time of HRV assessment, certain protocols were followed which included removing any metal objects from the body (including coins, jewelry), observing silence & relaxation with closed eyes, supine posture with palms facing upwards, and no physical movements. HRV recording room was maintained an ambient temperature of 22°C with no direct light exposure on the volunteer. After placement of electrodes, ECG was recorded using standard lead II electrodes and fed into a bio‐amplifier, and then digitized using an analog‐to‐digital converter. The data is then displayed on a computer with the data acquisition and analyzing software, AcqKnowledge 4.3+ (RRID:SCR_014279). BIOPAC MP150 system (RRID:SCR_014829) was used for ECG recording to measure HRV. We have taken HRV measurements for 13 min that include a 5‐min phase of supine relaxation, 15‐s tilting to 60°, 3 min tilted position (Gehrking et al., 2005), 15 s tilting back to re‐supine, and 5 min again in a supine relaxing position. From each ECG dataset, R‐R intervals were obtained, which were manually checked for any artifacts. Data during the transient period are removed from the analysis.

The commonly reported parameters of time‐domain (Camm et al., 1996; Sztajzel, 2004; Vanderlei et al., 2010; Table 1), frequency‐domain (Camm et al., 1996; Table 2), and non‐linear (Jeppesen et al., 2014; Shaffer & Ginsberg, 2017; Toichi et al., 1997; Table 3) were extracted using the Python HRV package.

**TABLE 1:** Time domain and geometric domain parameters of heart rate variability

Parameters	Description	Marker
SDNN (ms)	Standard deviation of NN intervals	Reflects total HRV
RMSSD (ms)	Square root of the mean squared differences of successive RRIs	Reflects vagal activity
pNN50	The proportion of NN50 divided by total number of NN intervals	Reflects vagal activity
Tidx	Triangular Index	Reflects overall HRV

Abbreviation: HRV, heart rate variability.

**TABLE 2:** Frequency domain parameters of heart rate variability

Parameters	Description	Marker
LF (0.04–0.15 Hz) (ms^2^)	Low frequency	Reflects both sympathetic and para‐ sympathetic activity. Generally indicates sympathetic activity
HF (0.15–0.4 Hz) (ms^2^)	High frequency	Reflects parasympathetic (vagal) activity
Total power (ms^2^)		Reflects over all HRV
LF/HF (ms^2^)		Reflects sympatho‐vagal Balance
LF (nu)	LF/TP‐VLF	Reflects sympathetic and parasympathetic activity
HF (nu)	HF/TP‐VLF	Reflects parasympathetic activity

Abbreviation: HRV, heart rate variability.

**TABLE 3:** Non‐linear parameters of heart rate variability

Parameters	Description	Marker
SD1	SD1 is a measure of the spread of RR intervals on the Poincaré plot perpendicular to the line of identity	Parasympathetic activity
SD2	SD2 is a measure of the spread of RR intervals on the Poincaré plot along the line of identity. It is an index of long‐term RR interval fluctuations	Sympathetic and parasympathetic activity
SDR	The ratio between short and long term fluctuations of the RR intervals	Vago‐sympathetic activity
CSI	The Cardiac Sympathetic Index (Toichi, 1997), calculated by dividing the longitudinal variability of the Poincaré plot (4*SD2) by its transverse variability (4*SD1)	Sympathetic activity
CVI	The Cardiac Vagal Index (Toichi, 1997), equal to the logarithm of the product of longitudinal (4*SD2) and transverse variability (4*SD1)	Parasympathetic activity
SampEn	The sample entropy measure of HRV	Measures overall HRV

Abbreviation: HRV, heart rate variability.

To observe the extent of HUT response in different *Prakriti* individuals, we have calculated the relative change of HRV parameters between supine to tilt (%*Δ*_1_) and also between tilt to resupine (%*Δ*_2_) positions. The relative change was calculated as follows:

$${\%∆}_{1}=\frac{\text{Tilt}\;\mathrm{HRV}\;\text{parameters}-\text{Supine}\;\mathrm{HRV}\;\text{parameters}}{\text{Supine}\;\mathrm{HRV}\;\text{parameters}}\times 100$$

$${\%∆}_{2}=\frac{\text{Resupine}\;\mathrm{HRV}\;\text{parameters}-\text{Tilt}\;\mathrm{HRV}\;\text{parameters}}{\text{Tilt}\;\mathrm{HRV}\;\text{parameters}}\times 100$$

To observe the age‐related contributions, we also compared HR and HRV parameters in two end of the spectrum age groups (lower age group, 18–20 years vs upper age group, 35–40 years) across *Prakriti*.

### 2.4 Statistical analysis

Each parameter was tested for distribution of the data based on the standard normality test, Shapiro–Wilk's test (*p* > 0.05). The following tests were used for comparison: One‐way ANOVA for normally distributed parameters and the Kruskal‐Wallis test for non‐normal distributed parameters. Paired *t*‐test and Wilcoxon were used for pairwise comparison of phase‐wise analysis. For *Prakriti*‐wise analysis as well as for age group‐based comparisons, *t*‐test and Wilcoxon were used for pairwise comparison. For post hoc analysis or multiple comparison correction, Bonferroni correction was used. *p* < 0.05 was considered statistically significant. All statistical analyses were performed using R software.

## 3 RESULTS

The study was conducted on 379 healthy individuals with no known medical problems. The mean age of the subjects was 27.3 ± 6.02 years and their BMI ranged from 22.21 ± 5.03 kg/m^2^.

The demographic details of the study participants at baseline level are listed in Table 4.

**TABLE 4:** Demographic details (*n* = 379)

Parameters	Characteristics
Gender (F/M)	217/162
Age (years)	27.3 ± 6.02
Height (cm)	160.32 ± 9.31
Weight (kg)	57.48 ± 15.53
BMI (kg/m^2^)	22.21 ± 5.03
Heart rate (BPM)	71.79 ± 11.5
Systolic blood pressure (mmHg)	110.75 ± 10.66
Diastolic blood pressure (mmHg)	71.32 ± 13.71

*Note*: Values are expressed as mean ± SD.

Abbreviations: BMI, body mass index; BPM, beats per minutes; cm, centimeter; F, female; kg, kilogram; M, male; mmHg, millimeters of mercury; y, years.

### 3.1 Effect of head‐up tilt on heart rate variability parameters at population level

HRV was assessed by the linear (time and frequency domain) and non‐linear methods. During HUT, the heart rate significantly increases. However, the extent of the increase is different for different individuals. We observed that all the parameters which signified the parasympathetic activity such as RMSSD, pNN50, HF, HF (nu) SD1, CV1 were significantly decreased. In contrast, we noted a substantial increase in sympathetic parameters such as LF (nu), CSI, SD2. Further, changes in the sample entropy also indicate a change in overall HRV upon tilt. Moreover, changes in LF/HF and SDR were also in line with the above sympathetic and parasympathetic changes that explain the significant changes in autonomic balance during the tilt phase. The HR & HRV alterations that occurred during the tilt phase were returned to that of the rest phase during the resupine position, with an even more parasympathetic activity observed in the resupine position when compared to the rest (Table 5).

**TABLE 5:** Heart rate variability indices during head‐up tilt at 60°

Parameters	Supine (*n* = 379)	Tilt (*n* = 379)	Re‐supine (*n* = 379)	*p*‐value
Heart rate (BPM)	70.78 (63.66–78.84)	84.15 (76.25–93.79)	70.2 (62.64–77.56)	1.74E‐53^aaaa,bbbb,cccc^
Time domain				
SDNN(ms)	46.56 (33.91–62.35)	45.75 (34.64–60.54)	58.55 (42.48–75.49)	1.46E‐15^bbbb,cccc^
RMSSD(ms)	42.95 (27.12–60.58)	22.72 (15.97–31.87)	46.15 (31.61–63.67)	6.64E‐57 ^aaaa,bbbb,cccc^
pNN50	22.16 (5.1–42.3)	2.78 (0.82–8.78)	25.91 (8.55–45.12)	2.16E‐52^aaaa,bbbb,cccc^
Geometric domain				
Tidx	11.2567 (9.1–14.93)	10.7 (8.26–13.38)	13.608 (10.44–17.54)	3.79E‐16^aaaa,bbbb,cccc^
Frequency domain				
LF (ms^2^)	444.77 (248.29–820.71)	459.03 (240.26–883.34)	481.13 (266.87–924.24)	0.277^b,c^
HF (ms^2^)	465.24 (213.54–990.74)	188.45 (90.81–360.79)	496.27 (226.42–985.76)	7.68E‐35^aaaa,bbbb,cc^
LF (nu)	49.53 (36.74–61.92)	67.99 (53.85–79.63)	48.88 (36.18–60.66)	1.54E‐41^aaaa,bbbb^
HF (nu)	46.43 (34.22–58.37)	27.91 (15.99–42.07)	46.61 (32.95–58.44)	6.00E‐38^aaaa,bbbb^
Total power (ms^2^)	1370.22 (739.72–2819.9)	1211.46 (680.71–2129.44)	1719.55 (919.22–2896.42)	6.20E‐06^aaaa,bbbb,cccc^
LF/HF	1.04 (0.63–1.82)	2.47 (1.31–4.78)	1.05 (0.64–1.87)	6.84E‐42^aaaa,bbbb^
Non‐linear analysis				
SD1 (ms)	30.26 (19.56–42.97)	16.3 (11.32–22.66)	32.58 (22.3–45.04)	8.94E‐57^aaaa,bbbb,cccc^
SD2 (ms)	56.69 (42.53–74.98)	61.21 (47.16–81.06)	73.81 (54.4–93.31)	4.68E‐15^aa,bbbb,cccc^
SDR	0.51 (0.41–0.65)	0.26 (0.21–0.36)	0.44 (0.35–0.56)	5.94E‐89^aaaa,bbbb,cccc^
CSI	1.95 (1.55–2.44)	3.81 (2.82–4.82)	2.25 (1.8–2.88)	8.52E‐34^aaaa,bbbb,cccc^
CVI	4.45 (4.13–4.69)	4.19 (3.96–4.45)	4.6 (4.3–4.82)	3.39E‐89^aaaa,bbbb,cccc^
SampEn	1.63 (1.47–1.82)	1.2 (0.93–1.46)	1.54 (1.36–1.74)	2.75E‐62^aaaa,bbbb,cccc^

Abbreviations: BPM, beats per minutes; CSI, Cardiac Sympathetic Index; CVI, Cardiac Vagal Index; HF, high frequency; LF, low frequency; LF/HF, LF and HF ratio; ms, millisecond; nu, normalized unit; pNN50, percentage of NN50; RMSSD, root mean square of successive R‐R interval differences; SampEn, sample entropy; SD1, standard deviation of instantaneous beat‐to‐beat variability; SD2, standard deviation of long‐term beat to‐beat variability; SDR, SD1/SD2 ratio; SDNN, standard deviation of normal to normal R‐R intervals; Tidx, Triangular Index.

^a^Supine compared to tilt; ^b^tilt compared to resupine; ^c^supine compared to resupine. *p < 0.05; **p < 0.01; ***p < 0.001; ****p < 0.0001; *∋{a,b,c}.

### 3.2 *Prakriti* segregation

Further, we have segregated these 379 healthy individuals into different *Prakriti* groups: *Vata* (*n* = 97), *Pitta* (*n* = 68), *Kapha* (*n* = 68), and Mixed (*n* = 146) with similar age (Table 6).

**TABLE 6:** Distribution of individuals into different *Prakriti* groups

Parameters	*Vata* (*n* = 97)	*Pitta* (*n* = 68)	*Kapha* (*n* = 68)	Mixed (*n* = 146)	*p*‐value
Gender (F/M)	70/27	28/40	36/32	83/63	
Age, years	27.76 ± 6.16	25.75 ± 5.81	29.09 ± 5.8	26.88 ± 5.95	
Height (cm)	157.59 ± 8.92	163.98 ± 9.98	161.72 ± 8.56	159.79 ± 8.98	
Weight (kg)	44.14 ± 7.05	60.69 ± 12.18	75.27 ± 12.28	56.57 ± 13.58	
BMI (kg/m^2^)	17.72 ± 1.93	22.42 ± 2.87	28.76 ± 3.86	22.03 ± 4.38	<2.2e‐16^aaaa,bbbb,cccc,dddd,eeee^
Systolic blood pressure (mmHg)	108.64 ± 8.91	109.42 ± 10.32	113.62 ± 10.33	111.39 ± 11.71	0.00159^aa,b^
Diastolic blood pressure (mmHg)	69.65 ± 14.29	70.12 ± 11.5	75.53 ± 7.89	71.01 ± 15.93	0.00136^aa,bb^

*Note*: Values are expressed as mean **±** SD.

Abbreviations: BMI, body mass index; BPM, beats per minutes; cm, centimeter; F, female; kg, kilogram; M, male; mmHg, millimeters of mercury; y, years.

^a^ *Vata* compared to *Kapha*; ^b^ *Kapha* compared to *Pitta*; ^c^ *Vata* compared to *Pitta*; ^d^ *Kapha* compared to Mixed; ^e^ *Vata* compared to Mixed. *p < 0.05; **p < 0.01; ***p < 0.001; ****p < 0.0001; *∋ {a,b,c,d,e}.

**FIGURE 1:**
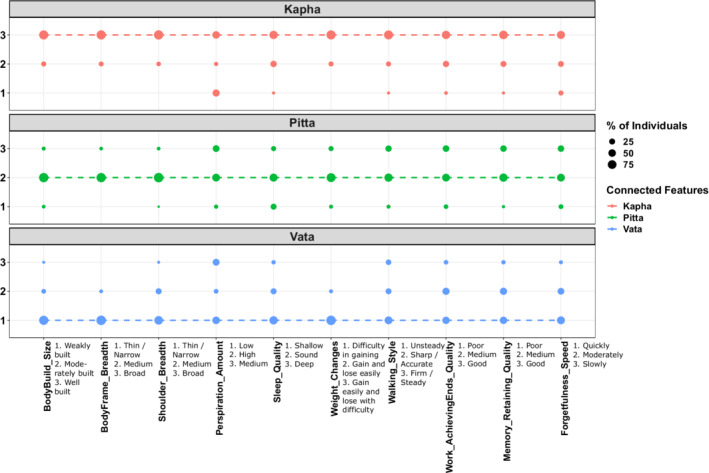
Representative set of phenotypes differentiating extreme Prakriti. Each dot represents the percentage of individuals with a particular feature value in each of the extreme *Prakriti* groups. The line connects values of all the features that correspond to same *dosha*, i.e. 1 for *Vata*, 2 for *pitta* and 3 for *Kapha* descriptions. As observed that extreme *Prakriti* individuals had higher proportion of individual feature values corresponding to respective *dosha*.

The phenotype data of *Prakriti* was analyzed for identification of extreme *Prakriti* individuals (*Vata, Pitta*, and *Kapha*) by the physicians. We also performed software‐based analysis using the models built earlier, to determine their *Prakriti* (Tiwari et al., 2017). The software results corroborate with the physician's *Prakriti* determination. Briefly, the *Prakriti* classification based on the questionnaire encompasses the anatomical, physiological, physical activity, and psychological phenotypes. These are captured through multiple features and each of them has values corresponding to *Vata, Pitta*, and *Kapha* descriptions. We observed that *Vata* individuals had thin and narrow‐body frame with weakly developed body build, shallow sleep, low perspiration levels, difficulty in gaining weight, quickly adapt to changes, and less tolerance for cold temperature etc. On the other hand, *Kapha* individuals had broad body frames with well‐developed body build, a tendency to gain weight easily, deep sleep, were less forgetful with good memory retention, preferred being less mobile, usually did not like frequent changes, and tendency to complete the tasks once undertaken etc. Similarly, *Pitta* individuals had sound sleep, higher perspiration levels, could gain as well as lose weight easily, sharp walking style, and less tolerance for hot temperatures etc (Figure 1).

The BMI was significantly higher in *Kapha* as compared to other groups. Furthermore, we also observed significantly higher SBP and DBP in *Kapha* as compared to *Vata* and *Pitta*.

For HRV parameters analysis we initially analyzed the cohort 1 and cohort 2 data separately and found that only heart rate was observed to be significantly higher in *Kapha* as compared to *Vata* at baseline in cohort 1. However, there was no significant difference in cohort 2 (Figures S1 and S2: all Supporting information is available at https://doi.org/10.14814/phy2.15435). But results in response to head‐up tilt were found to be similar in cohort 1 and cohort 2. Hence, we merged the data and performed the final analysis.

#### 3.2.1 *Prakriti‐*specific impact on heart rate variability

##### At supine phase (baseline)

**FIGURE 2:**
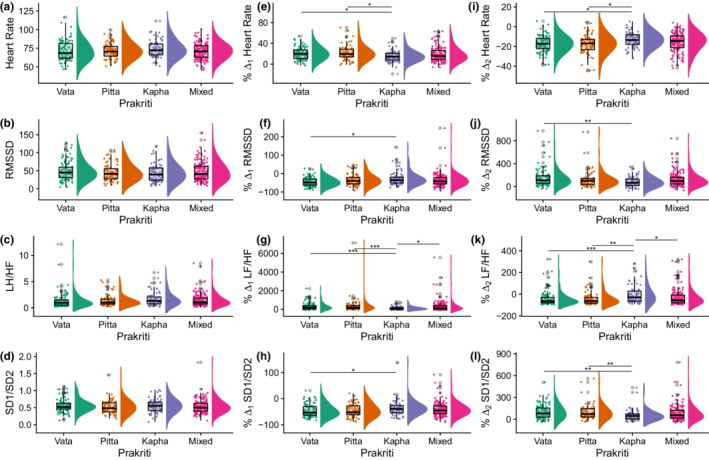
*Prakriti‐*specific differences in HR and HRV parameters: At baseline (a–d), relative change from supine to tilt (e–h), and relative change from tilt to resupine (i–l). bpm, beats per minutes; HR, heart rate; RMSSD, root mean square of successive R‐R interval differences; *p < 0.05; **p < 0.01; ***p < 0.001; ****p < 0.0001.

After segregation of all subjects into *Prakriti* groups, no significant differences were observed among *Prakriti* groups in heart rate and heart rate variability at baseline level after Bonferroni correction (Table 7; Figure 2). But without correction, we found HF was lower in *Kapha* as compared to *Vata* and Mixed group. Total HRV Power (TP) and SD2 were also lower in *Kapha* individuals as compared to *Pitta*. LF (nu) and HF (nu) were found to be high and low, respectively, in *Kapha* as compared to *Vata*. LF/HF ratio was also high in *Kapha* as compared to *Vata*. We also found Tidx was lower in *Kapha* as compared to *Vata* and *Pitta* (Table S1; Figures S3 and S4).

**TABLE 7:** Heart rate variability indices in different *Prakriti* groups at baseline (rest in supine position)

Parameters	*Vata* (*n* = 97)	*Pitta* (*n* = 68)	*Kapha* (*n* = 68)	Mixed (*n* = 146)	*p*‐value
Heart rate (bpm)	68.51 (61.65–81.43)	70.535 (64.4–77.45)	72.265 (66.06–80.82)	70.78 (62.93–78.42)	0.446
Time domain					
SDNN (ms)	48.52 (34.39–64.63)	47.36 (36.77–60.4)	42.14 (31.57–53.86)	45.905 (33.42–66.28)	0.237
RMSSD (ms)	44.99 (31.28–59.96)	41.42 (27.63–55.72)	39.63 (23.93–57.89)	41.315 (27.21–62.53)	0.568
pNN50	27.21 (8.35–41.03)	20.41 (5.61–37.69)	19.825 (2.95–39.69)	17.23 (4.81–45.07)	0.488
Geometric domain					
Tidx	11.21 (9.62–15.35)	11.73 (9.61–15.25)	10.4 (8.27–12.78)	11.475 (9.11–15.07)	0.085
Frequency domain					
LF (ms^2^)	424.48 (243.08–807.78)	518.805 (321.56–764.08)	380.67 (193.19–697.74)	493.38 (246.15–934.98)	0.348
HF (ms^2^)	482.2 (277.62–1061.76)	499.05 (245.38–826.18)	355.035 (125.32–729.7)	454.5 (197.84–1126.44)	0.101
LF (nu)	47.37 (29.91–59.66)	47.765 (40.58–59.96)	54.725 (42.11–66.27)	49.56 (38–63.2)	0.146
HF (nu)	49.64 (38.34–64.52)	47.145 (36.32–56.61)	42.615 (31.09–54.57)	46.91 (33.63–57.87)	0.105
Total power (ms^2^)	1495.8 (818.44–2868.4)	1417.51 (968.37–3117.31)	1088.895 (670.31–2136.67)	1476.91 (711.79–2833.51)	0.168
LF/HF	0.92 (0.47–1.49)	1 (0.7–1.63)	1.29 (0.75–2.05)	1.04 (0.7–1.85)	0.106
Non‐linear analysis					
SD1 (ms)	31.86 (22.14–42.44)	29.33 (19.83–39.47)	28.07 (16.85–40.93)	29.045 (19.29–44.87)	0.532
SD2 (ms)	58.86 (40.83–80.14)	58.895 (48.03–71.64)	52.195 (41.48–64.36)	56.325 (42.19–79.3)	0.127
SDR	0.52 (0.46–0.63)	0.485 (0.38–0.65)	0.555 (0.42–0.66)	0.5 (0.4–0.63)	0.297
CSI	1.91 (1.58–2.18)	2.065 (1.54–2.63)	1.795 (1.51–2.4)	2 (1.59–2.48)	0.307
CVI	4.48 (4.17–4.69)	4.46 (4.16–4.63)	4.35 (4.05–4.6)	4.465 (4.12–4.77)	0.302
SampEn	1.66 (1.48–1.82)	1.635 (1.47–1.84)	1.69 (1.46–1.85)	1.63 (1.45–1.78)	0.733

*Note*: Values are expressed as median (interquartile range).

Abbreviations: BPM, beats per minutes; CSI, Cardiac Sympathetic Index; CVI, Cardiac Vagal Index; HF, high frequency; LF, low frequency; LF/HF, LF and HF ratio; ms, millisecond; nu, normalized unit; pNN50, percentage of NN50; RMSSD, root mean square of successive R‐R interval differences; SampEn, sample entropy; SD1, standard deviation of instantaneous beat‐to‐beat variability; SD2, standard deviation of long‐term beat to‐beat variability; SDR, SD1/SD2 ratio; SDNN, standard deviation of normal to normal R‐R intervals; Tidx, Triangular Index.

In age‐wise comparison, we observed that there was a significantly lower heart rate in lower age group, 18–20 years (*n* = 67) age group as compared to upper age group, 35–40 years (*n* = 70). We also found significantly lower parasympathetic activity in terms of RMSSD, pNN50, HF, CVI, and SD1 and significantly lower overall HRV (SDNN, TP, and Tidx) in upper age group as compared to lower age group. Further to understand the effect of age on individual *Prakriti*, we analyzed the age group data for each *Prakriti*. We found that there was significantly lower parasympathetic (RMSSD, pNN50, HF, CVI, and SD1) and overall HRV (SDNN, TP, and Tidx) in *Kapha* individuals of upper age as compared to lower age group. Upper age group showed significantly lower HRV both in *Pitta* and *Vata* group (TP and TIDX) and Mixed group (SDNN, TP, and TIDX) (Table S7).

##### In response to head‐up tilt

The directionality of the change was found to be the same in all groups and we observed SDNN, LF, HF (nu), LF/HF, CSI, SD2, SDR, and SampEn were found to significantly differ among *Prakriti* in tilt position (Table S2; Figures S3 and S4). Further to see the extent of relative change in response to HUT it was necessary to look at the delta percentage change (%*Δ*_1_) between tilt and supine values. From HRV analysis in different *Prakriti* types in response to orthostatic stress, we observed that the relative change in heart rate was higher in *Vata and Pitta* individuals as compared to *Kapha* (Table 8). Also, we found the relative change in RMSSD, HF, HF (nu), and SD1 were significantly lower in *Kapha* as compared to *Vata* and the change in HF (nu) was also significantly lower as compared to *Pitta*. The changes in HF and HF (nu) were also significantly lower in *Kapha* as compared to the Mixed group. Both *Vata* and *Kapha* go down to the same level but *Kapha* has very minimal change. In contrast, the relative change in LF (nu) and CSI was significantly lower in *Kapha* as compared to *Vata* and LF (nu) as compared to *Pitta*. In line with the above observations, the relative change in LF/HF was significantly lower in *Kapha* as compared to *Vata*, *Pitta*, and Mixed group. Accordingly, the change in SDR was observed to be lower in *Kapha* as compared to *Vata*. The relative change in SampEn was lower in *Kapha* as compared to *Pitta* and Mixed group (Table 8; Figure 2).

**TABLE 8:** The relative change (%*Δ*_1_) of heart rate variability indices among different *Prakriti* groups in response to orthostatic stress than supine to tilt position

Parameters	*Vata* (*n* = 97)	*Pitta* (*n* = 68)	*Kapha* (*n* = 68)	Mixed (*n* = 146)	*p*‐value
%*Δ*_1_ heart rate	19.79 (10.85–27.35)	19.21 (13.89–28.52)	14.32 (7.86–20.56)	15.74 (9.13–26.14)	0.00841^a,b^
Time domain					
%*Δ*_1_ SDNN	−6.45 (−29.08–15.18)	4.69 (−18.14–39.54)	−0.77 (−17.16–16.92)	1.82 (−26.35–25.12)	0.08261
%*Δ*_1_ RMSSD	−48.29 (−62.67–29.84)	−40.32 (−56.13–21.02)	−36.02 (−51.33–19.38)	−41.79 (−56.42–21.55)	0.01888^a^
%*Δ*_1_ pNN50	−81.38 (−95.45–54.78)	−76.37 (−92.07–50.98)	−71.15 (−89.25–31.82)	−79.22 (−94.16–47.06)	0.18538
Geometric domain					
%*Δ*_1_ Tidx	−15.29 (−32.73–2.32)	−6.91 (−22.82–15.17)	−6.11 (−21.71–12.55)	−8.6 (−25.81–16.52)	0.08555
Frequency domain					
%*Δ*_1_ LF	−13.6 (−50.14–46.5)	36.68 (−37.47–95.59)	5.27 (−38.19–63.34)	−1.11 (−41.41–71.18)	0.15827
%*Δ*_1_ HF	−66.78 (−83.24–21.02)	−57.32 (−77.87–14.28)	−36.63 (−64.35–22.55)	−56.86 (−79.09–15.92)	0.00112^aaa,c^
%*Δ*_1_ LF (nu)	43.93 (10.07–84.17)	47.68 (14.28–70.71)	15.2 (−4.05–48.31)	28.82 (5.06–75.94)	0.0057^aa,b^
%*Δ*_1_ HF (nu)	−46.57 (−61.67–13.55)	−49 (−70.27–19.79)	−20.24 (−43.52–10.25)	−39.13 (−61.51–7.53)	0.00049^aa,bbb,c^
%*Δ*_1_ Total power	−19.72 (−53.12–28.98)	−11.61 (−42.51–57.53)	−9.6 (−43.26–50.08)	−12.43 (−46.74–31.01)	0.27394
%*Δ*_1_ LF/HF	195.37 (40.15–391.49)	204.52 (64.74–419.03)	57.4 (−13.08–180.52)	125.59 (16.42–390.85)	0.00047^aaa,bbb,c^
Non‐linear analysis					
%*Δ*_1_ SD1	−48.25 (−63.27–29.82)	−40.32 (−56.8–19.31)	−35.95 (−51.47–19.46)	−41.78 (−56.9–21.48)	0.01984^a^
%*Δ*_1_ SD2	1.39 (−19.64–27.42)	15.52 (−11.12–53.9)	10.58 (−8.79–34.24)	7.43 (−17.81–37.57)	0.1493
%*Δ*_1_ SDR	−53.45 (−63.64–30.3)	−52.15 (−61.57–27.79)	−39.32 (−53.76–26.67)	−45.49 (−57.49–28.7)	0.01539^a^
%*Δ*_1_ CSI	115.68 (45.27–176.02)	108.49 (39.44–155.67)	64.8 (36.06–117.13)	82.48 (39.96–136.61)	0.01478^a^
%*Δ*_1_ CVI	−5.96 (−10.16–2.25)	−4.27 (−8.88–1.15)	−2.88 (−7.64–0.51)	−4.01 (−9.93–0.35)	0.04365
%*Δ*_1_ SampEn	−29.7 (−41.83–14.19)	−37.48 (−50.35–16.09)	−18.01 (−34.49–6.85)	−19.03 (−38.04–6.78)	0.00042^bb,dd^

*Note*: Values are expressed as median (interquartile range).

Abbreviations: BPM, beats per minutes; CSI, Cardiac Sympathetic Index; CVI, Cardiac Vagal Index; HF, high frequency; LF, low frequency; LF/HF, LF and HF ratio; ms, millisecond; nu, normalized unit; pNN50, percentage of NN50; RMSSD, root mean square of successive R‐R interval differences; SampEn, sample entropy; SD1, standard deviation of instantaneous beat‐to‐beat variability; SD2, standard deviation of long‐term beat to‐beat variability; SDNN, standard deviation of normal to normal R‐R intervals; SDR, SD1/SD2 ratio; Tidx, Triangular Index.

^a^ *Vata* compared to *Kapha*; ^b^ *Kapha* compared to *Pitta*; ^c^ *Kapha* compared to Mixed; ^d^ *Pitta* compared to Mixed. *p < 0.05; **p < 0.01; ***p < 0.001; ****p < 0.0001; *∋ {a,b,c,d}.

Further to analyze the effect of age on HUT in *Prakriti‐*specific manner, we observed the relative change from supine to tilt was significantly less in the upper age group as compared to lower in HR, RMSSD, HF, HF (nu), SD1, LF, LF (nu), CSI, LHR, SDR, SampEn. But, contrary to baseline no significant differences were observed in *Kapha Prakriti* group among these extreme age groups. Whereas the orthostatic stress response was significantly lower in the upper age group of *Pitta* group (LF (nu), HF (nu), LHR), *Vata* (LF (nu), HF (nu), LHR, and SampEn) and mixed *Prakriti* group (HR, RMSSD, LF (nu), HF (nu), and LHR) (Table S8).

##### Recovery after HUT as observed during resupine phase

Although in all the three groups the HRV values were restored to the baseline values, the restoration or resupine values varied between *Prakriti* groups. We found significant differences in HF, HF (nu), and Tidx values between the groups in the resupine phase (Table S3; Figures S3 and S4). Further to see the extent of relative change post HUT, we calculated the delta percentage change (%*Δ*_2_) between the tilt values to resupine. The relative change in HR was lower in *Kapha* when compared to *Vata and Pitta*. The change in RMSSD, pNN50, HF, HF (nu), and SD1 were lower in *Kapha* as compared to *Vata*. Also, the change in HF and HF (nu) was lower in *Kapha* as compared to Mixed *Prakriti*. In the case of CSI and LF (nu), it was observed that the change is less in *Kapha* when compared to *Vata*. Moreover, the relative change in LF/HF and SDR in *Kapha* were lower than *Vata* and also the *Kapha* group showed lower SDR as compared to Mixed group. The relative change in SampEn was lower in *Kapha* and Mixed groups as compared to *Pitta* (Table 9; Figure 2).

**TABLE 9:** The relative change (%*Δ*_2_) of heart rate variability indices among different *Prakriti* groups during recovery response than tilt to resupine

Parameters	*Vata* (*n* = 97)	*Pitta* (*n* = 68)	*Kapha* (*n* = 68)	Mixed (*n* = 146)	*p*‐value
%*Δ*_2_ heart rate	−17.38 (−22.3–11.83)	−17 (−23.41–12.84)	−13.56 (−17.87–8.25)	−14.49 (−21.39–9.44)	0.00832^a,b^
Time domain					
%*Δ*_2_ SDNN	32.96 (7.38–63.49)	22.99 (−15.86–44.95)	27.38 (−3.33–62.33)	27.07 (1.43–67.54)	0.13422
%*Δ*_2_ RMSSD	111.18 (57.87–189.77)	100.73 (37.49–143.42)	66.35 (20.21–130.45)	96.86 (45.94–164.32)	0.00919^aa^
%*Δ*_2_ pNN50	524.43 (196.75–1884.09)	255.16 (106.51–1184.62)	259.68 (50.78–545.56)	405.31 (100.89–1226.5)	0.03158^a^
Geometric domain					
%*Δ*_2_ Tidx	30.89 (8.43–67.46)	28.48 (−2.72–54.03)	18.67 (−0.83–44.54)	28.44 (2.45–57.86)	0.25086
Frequency domain					
%*Δ*_2_ LF	21.52 (−21.85–105.15)	5 (−56.03–84.19)	8.55 (−22.06–70.32)	11.44 (−37.19–91.09)	0.46756
%*Δ*_2_ HF	234.76 (76.57–468.42)	195.29 (48.26–368.41)	67.41 (−1.15–228.86)	145.09 (36.07–428.32)	0.0003^aaa,b,c^
%*Δ*_2_ LF(nu)	−31.25 (−49.35–10.21)	−27.12 (−49.99–14.02)	−12.38 (−33.42–4.79)	−23.5 (−41.74–4.65)	0.0025^aa,bb^
%*Δ*_2_ HF(nu)	79.84 (12.28–152.4)	96.1 (11.24–219.8)	18.31 (−14.12–81.8)	67.39 (2.61–181.53)	0.00097^aa,b,c^
%*Δ*_2_ Total power	66.29 (−2.35–132.6)	34.26 (−26.78–104.62)	44.96 (−28.99–85.53)	30.89 (−10.81–105.4)	0.28432
%*Δ*_2_ LF/HF	−64.49 (−79.67–29.17)	−62.79 (−82.68–28.66)	−29.88 (−64.05–28.72)	−56.84 (−79.81–8.4)	0.00049^aaa,bb,c^
Non‐linear analysis					
%*Δ*_2_ SD1	110.95 (57.53–187.8)	99.97 (33.95–143.61)	66.51 (23.18–131.42)	96.8 (44.49–164.84)	0.00987^aa^
%*Δ*_2_ SD2	21.95 (−0.26–49.97)	13.61 (−19.41–42.82)	17.72 (−10.93–53.89)	19.14 (−4.76–59.32)	0.2355
%*Δ*_2_ SDR	81.82 (30–155.56)	73.68 (31.22–146.37)	45.76 (5.48–73.81)	55.09 (17.24–123.77)	0.00168^aa,bb^
%*Δ*_2_ CSI	−44.03 (−61–23.12)	−42.19 (−59.64–23.07)	−31.49 (−41.71–6.47)	−35.4 (−55.41–13.76)	0.00178^aa,b^
%*Δ*_2_ CVI	9.68 (5.13–14.71)	8.64 (1.09–13.32)	7.83 (1.44–12.5)	8.95 (3.7–14.87)	0.06078
%*Δ*_2_ SampEn	31.25 (11.11–60)	44.72 (11.76–94.66)	17.5 (−7.37–52.57)	17.41 (−1.23–54.48)	0.0013^b,dd^

*Note*: Values are expressed as median (interquartile range).

Abbreviations: BPM, beats per minutes; CSI, Cardiac Sympathetic Index; CVI, Cardiac Vagal Index; HF, high frequency; LF, low frequency; LF/HF, LF and HF ratio; ms, millisecond; nu, normalized unit; pNN50, percentage of NN50; RMSSD, root mean square of successive R‐R interval differences; SampEn, sample entropy; SD1, standard deviation of instantaneous beat‐to‐beat variability; SD2, standard deviation of long‐term beat to‐beat variability; SDR, SD1/SD2 ratio; SDNN, standard deviation of normal to normal R‐R intervals; Tidx, Triangular Index.

^a^ *Vata* compared to *Kapha*; ^b^ *Kapha* compared to *Pitta*; ^c^ *Kapha* compared to Mixed; ^d^ *Pitta* compared to Mixed; *p < 0.05; **p < 0.01; ***p < 0.001; ****p < 0.0001; *∋ {a,b,c,d}.

Furthermore, in age‐wise analysis, we observed that the trend of HRV response from tilt to resupine was lower in upper age group. We observed that irrespective of *Prakriti*, HR and HRV parameters had significantly lower changes in upper age group. *Kapha* individuals showed no significant differences among age groups in tilt‐ to resupine except in SDR and CSI (Table S9).

**FIGURE 3:**
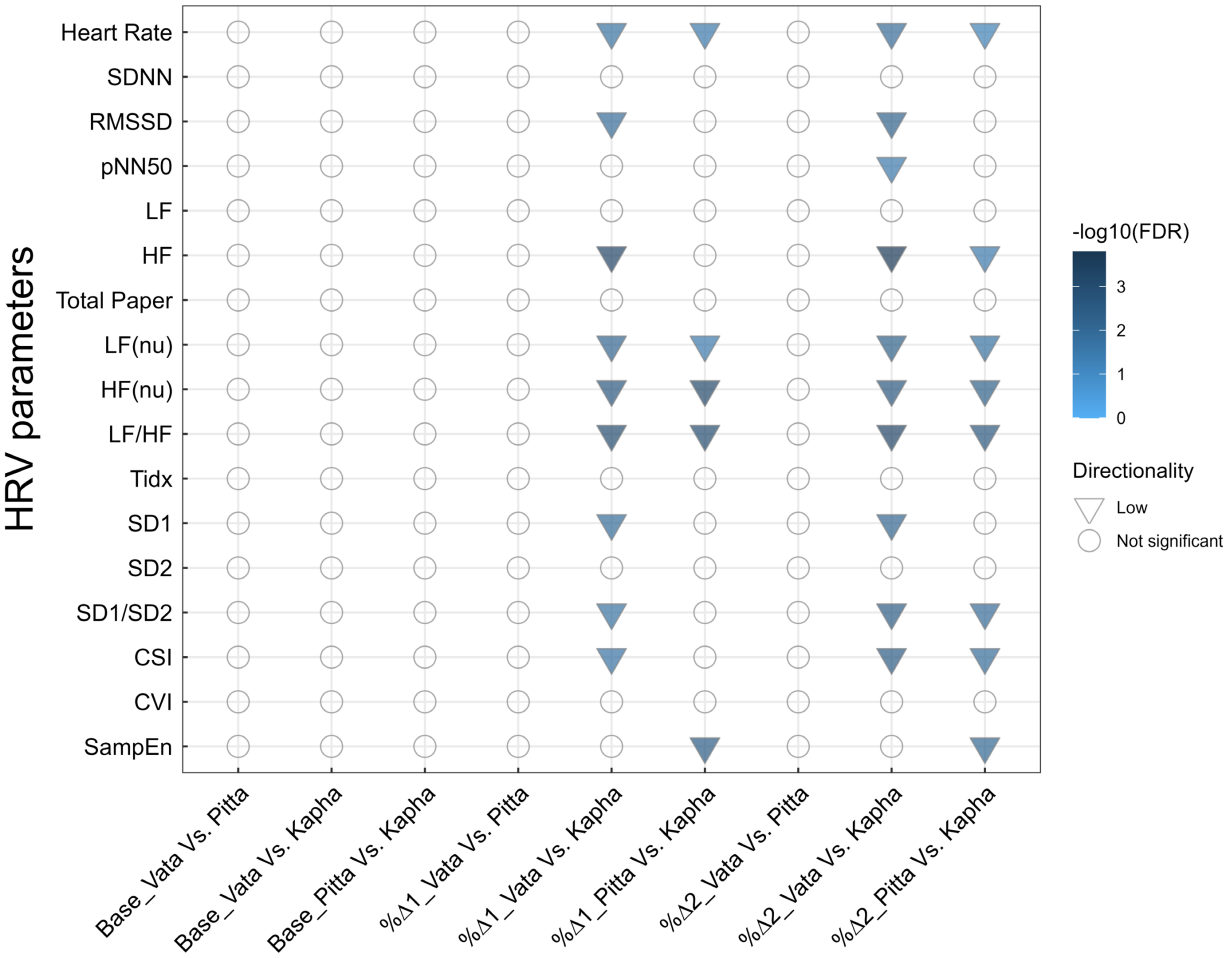
Overall representation of differences of HR and HRV parameters in *Prakriti‐*specific manner showing significance level and directionality in different stages of HUT test. Directionality is for first group as compared to second group. Base, baseline; CSI, cardiac sympathetic index; CVI, cardiac vagal index; HF, high frequency; LF, low frequency; LF/HF, LF and HF ratio; nu, normalized unit; pNN50, percentage of NN50; RMSSD, root mean square of successive R‐R interval differences; SampEn, sample entropy; SD1, standard deviation of instantaneous beat‐to‐beat variability; SD2, standard deviation of long‐term beat to‐beat variability; SDNN, standard deviation of normal to normal R‐R intervals; Tidx, triangular index; %∆1, relative change from supine to tilt; %∆2, relative change from tilt to resupine.

Overall, we observed that HR & HRV parameters do not significantly differ among *Prakriti* groups at the baseline levels. However, when challenged with HUT, *the Kapha Prakriti* group tends to have minimal change in HRV whereas *Vata* has a higher drop in parasympathetic activity‐related parameters such as HF, and RMSSD (Figure 3).

Furthermore, we also segregated and analyzed the mixed group (*n* = 146) into subgroups, *Vata‐Pitta* (*n* = 82), *Kapha‐Pitta* (*n* = 51), *Vata‐Kapha* (*n* = 13). We observed that there were no significant differences at baseline. However, we found that the relative change from supine to tilt in Heart Rate, and HRV was significantly lower in *Kapha‐Pitta* as compared to *Vata‐Pitta*. The change in parasympathetic activity (RMSSD, HF, HF(nu), SD1, and CVI) was higher in *Vata‐Pitta* as compared to *Kapha‐Pitta*. Similarly, the change in LF(nu), Tidx, and LHR was lower in *Kapha‐Pitta* than *Vata‐Pitta* and the change in HR and pNN50 was also significantly lower in *Vata‐Kapha* than *Vata‐Pitta* (Tables S4–S6).

To determine the significance and specificity of our observations in *Prakriti* groups, we generated 10,000 permuted datasets by removing *Prakriti* labels. A similar statistical analysis (Kruskal‐Wallis) was carried out among groups to calculate the difference in parameters of HRV (Prasher et al., 2008). We observed that the original group *p*‐value lies within the top 5% of the *p*‐values of 10,000 permuted datasets after sorting (Figure S5).

## 4 DISCUSSION

In this study, we report the effect of HUT on healthy individuals among two geographically distinct populations. We found significant differences in the time domain, frequency domain, and non‐linear parameters of HRV, indicating the sympathetic dominance and parasympathetic withdrawal during the orthostatic head‐up tilt test.

It is reported that HF power is decreased, LF/HF ratio increased significantly in response to HUT in the young subjects aged 23–39 years. Similar observations were also found in our study as we observed Heart Rate significantly increased and Parasympathetic parameters such as RMSSD, pNN50, HF, HF(nu), and SD1 decreased. Further, we also observed that LF/HF ratio increased after orthostatic stress in this study. They explained that an increase in the LF/HF ratio of HRV majorly depends on parasympathetic deactivation. So, in response to an orthostatic challenge, the general response is parasympathetic deactivation at the cardiac level and an increase in sympathetic activity is characterized by a decrease in HRV (Laitinen et al., 2004).

Poincare plot is the visualization of successive RR intervals in the Cartesian plane. The SD1 reflects parasympathetic activity and SD2 reflects sympathetic modulation on the sinus node (Mourot et al., 2004; Shaffer & Ginsberg, 2017). Our result of a decrease in SD1 during tilt position is consistent with previous results (Karmakar et al., 2011; Pawłowski et al., 2021). Likewise, CSI represents the cardiac sympathetic activity which also increased in tilt position & CVI which represents cardiac parasympathetic activity decreased in the tilt position. Thus, our observed responses were in the line with earlier reported studies.

Inter‐individual variability in response to HUT test was reported earlier in healthy individuals (Natale et al., 1995; Petersen et al., 2000). Here we show for the first time that healthy individuals stratified into extreme *Prakriti* groups exhibit differences in HRV parameters in response to HUT. The resting 5 min supine HRV parameters were not significantly different among the *Prakriti* groups after Bonferroni correction. As our study is only on healthy individuals of 18–40 years, it may be expected that no significant changes in 5‐min supine HRV would be observed. Although without correction, we found HF and HF (nu) which signifies the parasympathetic activity was lower in *Kapha* as compared to the *Vata* group at the baseline level. LF (nu) and LF/HF were also high in *Kapha* as compared to *Vata*.

We also found Tidx and TP were lower in *Kapha* as compared to *Pitta. In our* previous study, we reported that *Kapha* individuals have higher cholesterol, triglycerides, and LDL compared to other *Prakriti* groups albeit within the normal range (Prasher et al., 2008). Further, the previous studies have also shown HRV and lipid profile correlation, such as an increased triglyceride was associated with a decreased SDNN, increased level of LDL and total cholesterol were associated with decreased RMSSD and HF (nu). These findings and our present results together might suggest that *Kapha* group individuals, who have higher sympatho‐vagal balance and lower vagal tone could be susceptible to cardiovascular diseases.

During HUT, the direction of the relative change of time domain, frequency domain, geometrical and non‐linear parameters of HRV were identical among *Prakriti* groups. However, the magnitude of difference tends to significantly differ among *Prakriti* groups. As our study showed variability in response to orthostatic stress among healthy individuals when classified into different *Prakriti* groups, these results can also be useful while studying various diseases which are found to have orthostatic intolerance like Postural Orthostatic Tachycardia Syndrome (POTS), Vasovagal Syncope and also the recent COVID‐19 infections. Particularly, recent studies have shown that the COVID positive group was found to have higher overall HRV parameters of parasympathetic tone (RMSSD, pNN50, and HFms) when compared with the Non‐COVID group at baseline (Stute et al., 2021). Although when COVID positive individuals were subjected to an orthostatic challenge, they observed a higher drop in parasympathetic activity as compared to Non‐COVID individuals (Stute et al., 2021). In our study we have observed that the *Vata Prakriti* group showed a significantly higher relative change in HR, RMSSD of the time‐domain parameter as compared to *Kapha* in response to tilt. The frequency‐domain parameters like HF, HF (nu), LF (nu), and LF/HF ratio also showed a significantly higher relative change in the *Vata Prakriti* group. The trends similar to the *Vata* group are also reported in earlier studies, where patients with POTS showed, decreased parasympathetic tone. The HF attenuated significantly more in the POTS group at the beginning of HUT exposure and LF, increased significantly in the POTS group. Further, they also showed that LF/HF increased significantly in both groups in an upright position, but the increase was steeper in the POTS group (Orjatsalo et al., 2020). The non‐linear parameters such as SD1, SDR ratio, CSI, CVI, sample entropy were also showing a significantly higher relative change in the *Vata Prakriti* group. This indicates that COVID and POTS related orthostatic intolerance could be a *Vata Vikriti* condition. Although it can arise in any *Prakriti* type individuals, *Vata Prakriti* may be more susceptible. Our results are also in line with the Ayurveda descriptions of *Vata* and *Kapha Prakriti*, wherein *Vata* is described to be quick/ brisk in responding to stress whereas slow response and stability are hallmarks of *Kapha*. But further studies need to explore the mechanism to understand the differences in responses among healthy individuals and in COVID‐like diseases.

As regards relative change (%*Δ*_2_) of HRV parameters from tilt to re‐supine, *Kapha Prakriti* also showed a significantly lower relative change in HR, RMSSD, pNN50 of time‐domain parameters similar to what was observed in %*Δ*_1_. In frequency domain parameters, a significantly lower relative change was observed in HF, HF (nu), LF (nu) & LF/HF. In non‐linear parameters, the SD1, SDR, CSI & sample entropy showed significantly lower relative change. Baseline differences in HRV in relation to BMI have been reported earlier where it is shown significantly higher HF in individuals with <20 kg/m^2^ BMI (Molfino et al., 2009) and a significantly higher LF (nu) in overweight individuals compared with normal weight and underweight (Krishna et al., 2013). Also, the overweight individuals showed higher LF and lower HF in absolute values at baseline (Krishna et al., 2013; Molfino et al., 2009; Pal et al., 2012). Our observations of baseline HRV are in line where we also observed low BMI and high HF in the *Vata* group; high BMI, and low HF in *Kapha* group, although not statistically significant after Bonferroni correction. It would be worthwhile to mention that no study has been reported with such relation of HRV response to HUT and BMI or body weight. Because age is one of the factors that is already known to affect HRV (decreases with age) (Garavaglia et al., 2021; Umetani et al., 1998), when we looked for age‐dependent responses among *Prakriti* groups, we found that age‐related changes in response to HUTT were present in *Pitta* and *Vata Prakriti* groups. Whereas interestingly, the response in *Kapha Prakriti* remains constant even in extreme age groups. Because our study demonstrated that the response to orthostatic stress differed between *Prakriti* groups and also depended on age, we conclude that the response to orthostatic stress varies with age only in certain *Prakriti* types. Therefore, *Prakriti* stratification may help distinguish between people who are differentially predisposed to vasovagal syncope and more so with increasing age. Triggers for vasovagal syncope include orthostatic stress (changes in body position), heat exposure, and other forms of stress such as mental or physiological stress (Wieling et al., 2004; Task Force for the Diagnosis and Management of Syncope, European Society of Cardiology (ESC), et al., 2009). Hence, it would also be worthwhile to see how different *Prakriti* individuals exhibit differential sensitivity to these triggers. In addition, the *Prakriti* stratification may also be useful for guiding the treatment strategies.

The comparison between mixed *Prakriti* groups among themselves shows that the relative change from supine to tilt in HR and HRV is lower in *Kapha‐Pitta* as compared to *Vata‐Pitta*. These observations are similar to our above observations in dominant *Prakriti* groups as we can see here that *Prakriti* with *Kapha*‐*Pitta* dominance is less responsive as compared to *Vata*‐*Pitta* to orthostatic stress. This is the first study reporting variability among healthy individuals classified by the *Prakriti* method toward HUT response and changes during re‐supine.

Human health is the manifestation of a constant relationship between the human body and its surroundings. Increasing data reveal that healthy people have underlying molecular heterogeneity, which results in diverse responses to the environment, diseases, and treatments. Personalized medicine or the practice of deep phenotyping of an individual using multi‐system attributes that allow uncovering of underlying physiological variability and accordingly advocates precision interventions, is a necessity. HR and HRV responses to Head‐Up Tilt, are also known to have inter‐individual differences. In Ayurveda, the concept of *Prakriti* or an individual intrinsic constitution is strongly ingrained in personalized health management (Dey & Pahwa, 2014; Prasher et al., 2008, 2017).

## 5 CONCLUSIONS

In the present study, orthostatic challenge‐induced HRV changes were used to decipher the possible differences in different types of individuals. The results show that HR & HRV parameters do not significantly differ among *Prakriti* groups at the supine position. However, when challenged with HUT, *the Kapha Prakriti* group tends to have minimal change in HRV whereas *Vata* has a higher drop in parasympathetic activity as observed in POTS and also recently reported in COVID‐19 patients. Furthermore, the results indicate that cardiac autonomic modulation evaluated by standard HRV recording in different positions clearly delineates the differences within healthy populations segregated by Ayurveda *Prakriti* methods. The lower relative change (%*Δ*_1&2_) in various HRV parameters observed in the healthy *Kapha Prakriti* and a steeper decline in parasympathetic activity in *Vata* after inducing HUT may suggest susceptibility to autonomic dysfunction and associated conditions; however, further longitudinal studies with additional parameters are needed to explore the mechanism and allow predictive modeling for *Prakriti* based on HRV response.

## AUTHOR CONTRIBUTIONS

BP, and MM conceived and designed the study. TP and KKD designed the HRV protocol. PR, BKK, AK, BG, AS, TP, RR, DSP, DP, BP, and SaJ performed volunteer recruitment, *Prakriti* stratification and clinical phenotyping. BaP, RR, and PR performed HRV data analysis. RR, PR, BaP, and BP wrote the manuscript. BP, BaP, MM, and KKD reviewed the data and manuscript. All authors have read and approved the manuscript.

## FUNDING INFORMATION

The work was supported by a grant (MLP901) from the Council of Scientific and Industrial Research (CSIR) Govt. of India and M/o AYUSH for CoE Applied development in Ayurvedic *Prakriti* and Genomics (GAP0183) to BP and MM.

## CONFLICT OF INTEREST

The authors declare that the research was conducted in the absence of any commercial or financial relationships that could be construed as a potential conflict of interest.

## ETHICS STATEMENT

This study was carried out in accordance with the recommendations of the Indian Council of Medical Research, India guidelines for biomedical research, with written informed consent from all subjects. All subjects gave written informed consent in accordance with the Declaration of Helsinki. The protocol was approved by the Institutional Human ethics committees of K.E.M. Hospital and Research Centre, Pune as well as the Institute of Genomics and Integrative Biology, Delhi, India, and All India Institute of Medical Sciences, New Delhi.

## Supporting information


Figures S1–S5
Tables S1–S6

Tables S7–S9
