# Supplementary material for: Heart rate variability during head‐up tilt shows inter‐individual differences among healthy individuals of extreme Prakriti types
Source: Physiol Rep. 2022 Sep 15;10(17):e15435. doi: 10.14814/phy2.15435 (PMC9475339; doi:10.14814/phy2.15435)
Supplement: Supplementary file 1 — Figures S1–S5 Tables S1–S6 [file PHY2-10-e15435-s001.pdf]

Supplementary Information for

Heart Rate Variability during Head-Up Tilt shows inter-individual differences among healthy individuals of extreme *Prakriti* types

Ritu Rani[1,2,3,4]†, Prathiban Rengarajan[6]†, Tavpritesh Sethi[3,7], Bharat Krushna Khuntia[2,3], Arvind Kumar[2,3], Deep Shikha Punera[1,2,3,4], Deepika[1,2,3,], Bhushan Girase[5], Ankita Shrivastava[5], Sanjay K. Juvekar[5], Bala Pesala[8], Mitali Mukerji[1,2,3,8*], KK Deepak[6*], Bhavana Prasher[1,2,3,4*]

Affiliations

[1] Centre of Excellence for Applied Development of Ayurveda *Prakriti* and Genomics, CSIR-Institute of Genomics & Integrative Biology, Delhi, India

[2] CSIR's Ayurgenomics Unit–TRISUTRA (Translational Research and Innovative Science ThRough Ayurgenomics) CSIR-Institute of Genomics and Integrative Biology, New Delhi, India;

[3] Genomics and Molecular Medicine, CSIR-Institute of Genomics & Integrative Biology, Mathura Road, Delhi, India

[4] Academy of Scientific and Innovative Research, Ghaziabad, Uttar Pradesh, India.

[5] Vadu Rural Health Program, KEM Hospital Research Centre, Pune, India

[6] Department of Physiology, All India Institute of Medical Sciences, Ansari Nagar, New Delhi, India;

[7] Indraprastha Institute of Information Technology, Delhi, India, (Current address)

[8] Indian Institute of Technology Jodhpur, NH 62, Karwar, Jodhpur, Rajasthan, India (Current address)

[*] Correspondence: bhavana.p@igib.res.in; kkdeepak@gmail.com; mitali@iitj.ac.in;

† These authors have contributed equally to this work and share first authorship

Supplementary figures

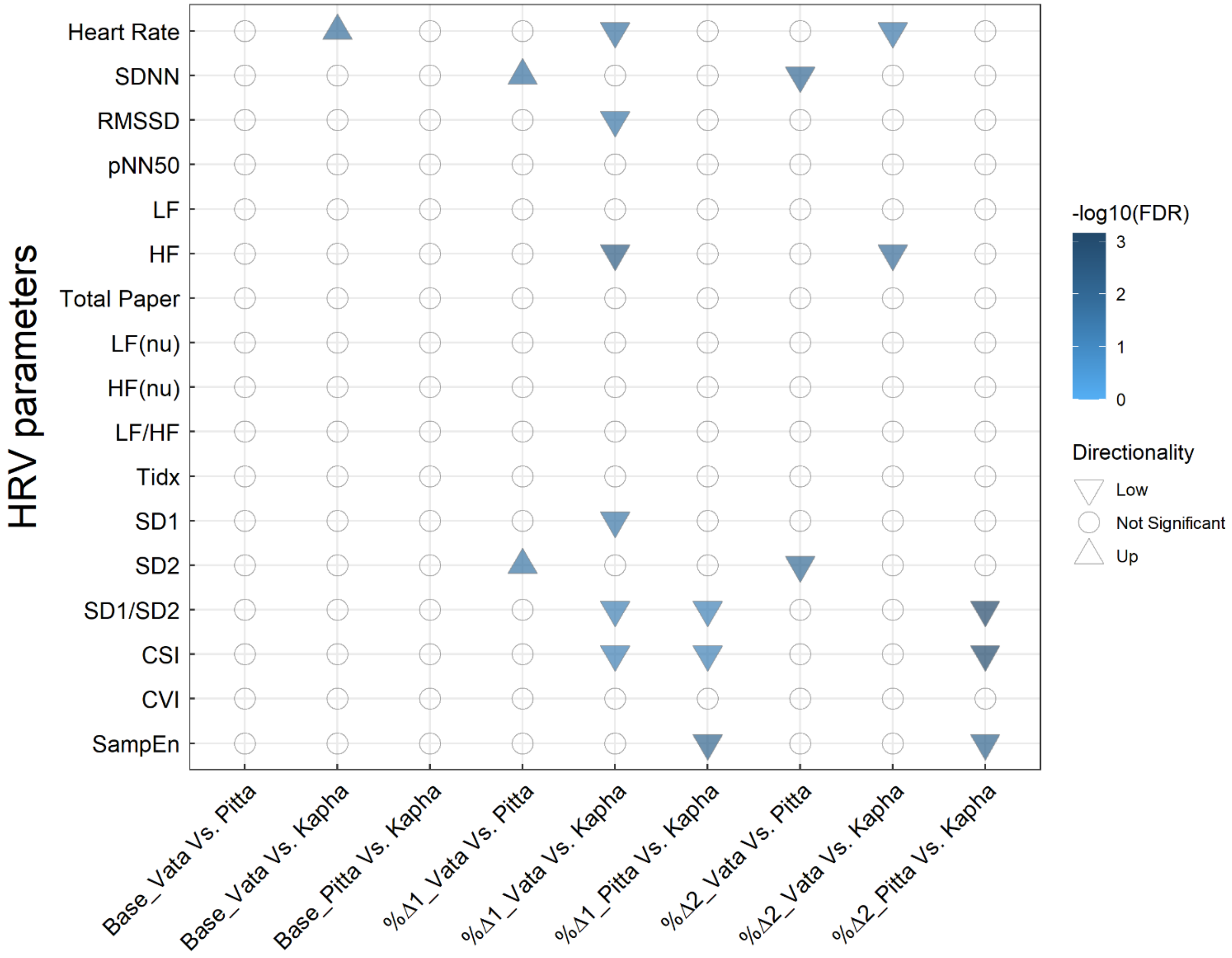

Figure S1: Overall representation (of Cohort 1) of differences of HR and HRV parameters in *Prakriti* specific manner showing significance level and directionality in different stages of HUT test. Directionality is for first group as compared to second group

Abbreviations: Base, Baseline; CSI, Cardiac Sympathetic Index; CVI, Cardiac Vagal Index; HF, high frequency; LF, low frequency; LF/HF, LF and HF ratio; nu, normalized unit; pNN50, percentage of NN50; RMSSD, root mean square of successive R-R interval differences; SampEn, Sample Entropy; SD1, standard deviation of instantaneous beat-to-beat variability; SD2, standard deviation of long-term beat to-beat variability; SDNN, standard deviation of normal to normal R-R intervals; Tidx, Triangular Index; %Δ1, relative change from supine to tilt; %Δ2, relative change from tilt to resupine.

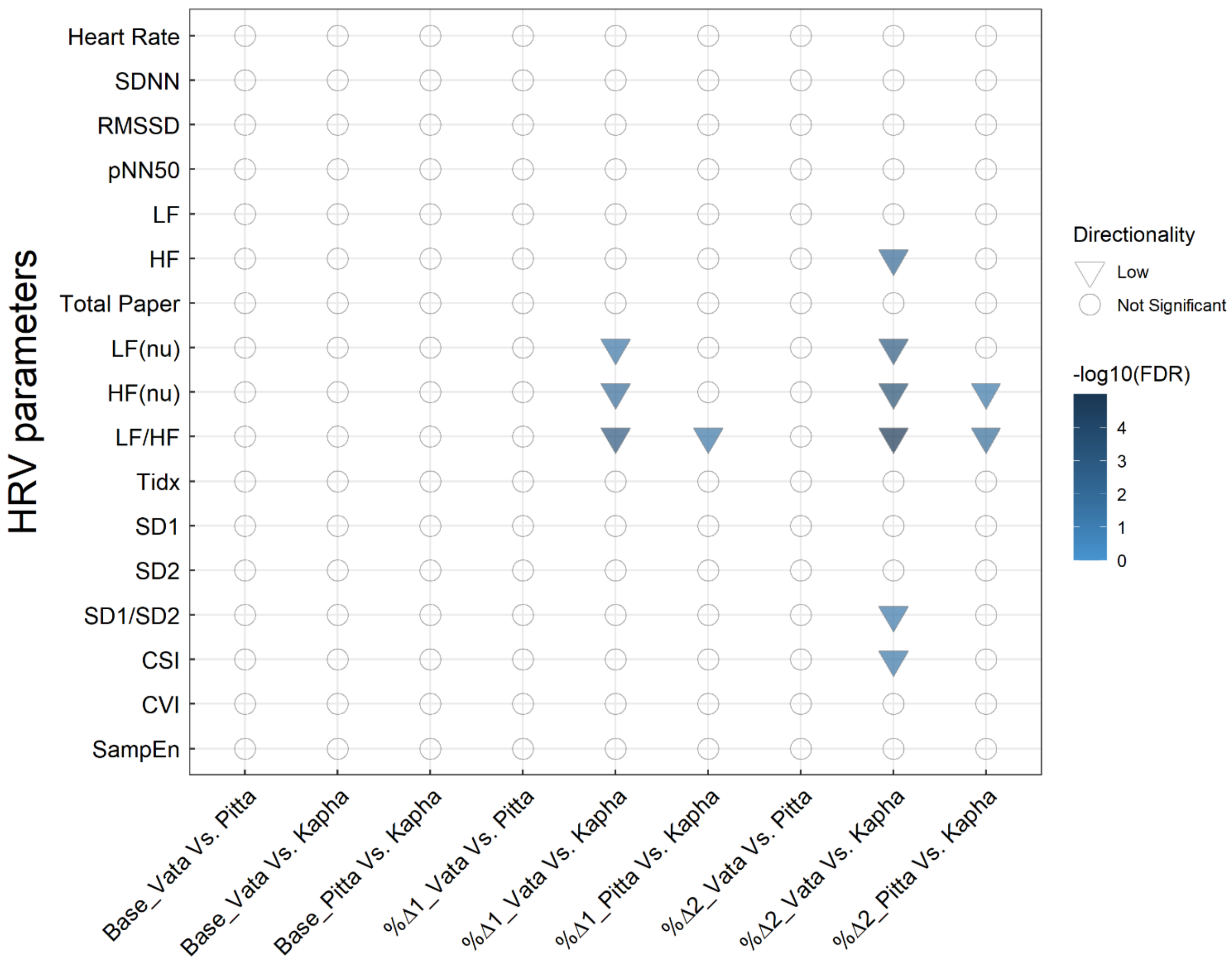

Figure S2: Overall representation (of Cohort 2) of differences of HR and HRV parameters in *Prakriti* specific manner showing significance level and directionality in different stages of HUT test. Directionality is for first group as compared to second group

Abbreviations: Base, Baseline; CSI, Cardiac Sympathetic Index; CVI, Cardiac Vagal Index; HF, high frequency; LF, low frequency; LF/HF, LF and HF ratio; nu, normalized unit; pNN50, percentage of NN50; RMSSD, root mean square of successive R-R interval differences; SampEn, Sample Entropy; SD1, standard deviation of instantaneous beat-to-beat variability; SD2, standard deviation of long-term beat to-beat variability; SDNN, standard deviation of normal to normal R-R intervals; Tidx, Triangular Index; %Δ1, relative change from supine to tilt; %Δ2, relative change from tilt to resupine.

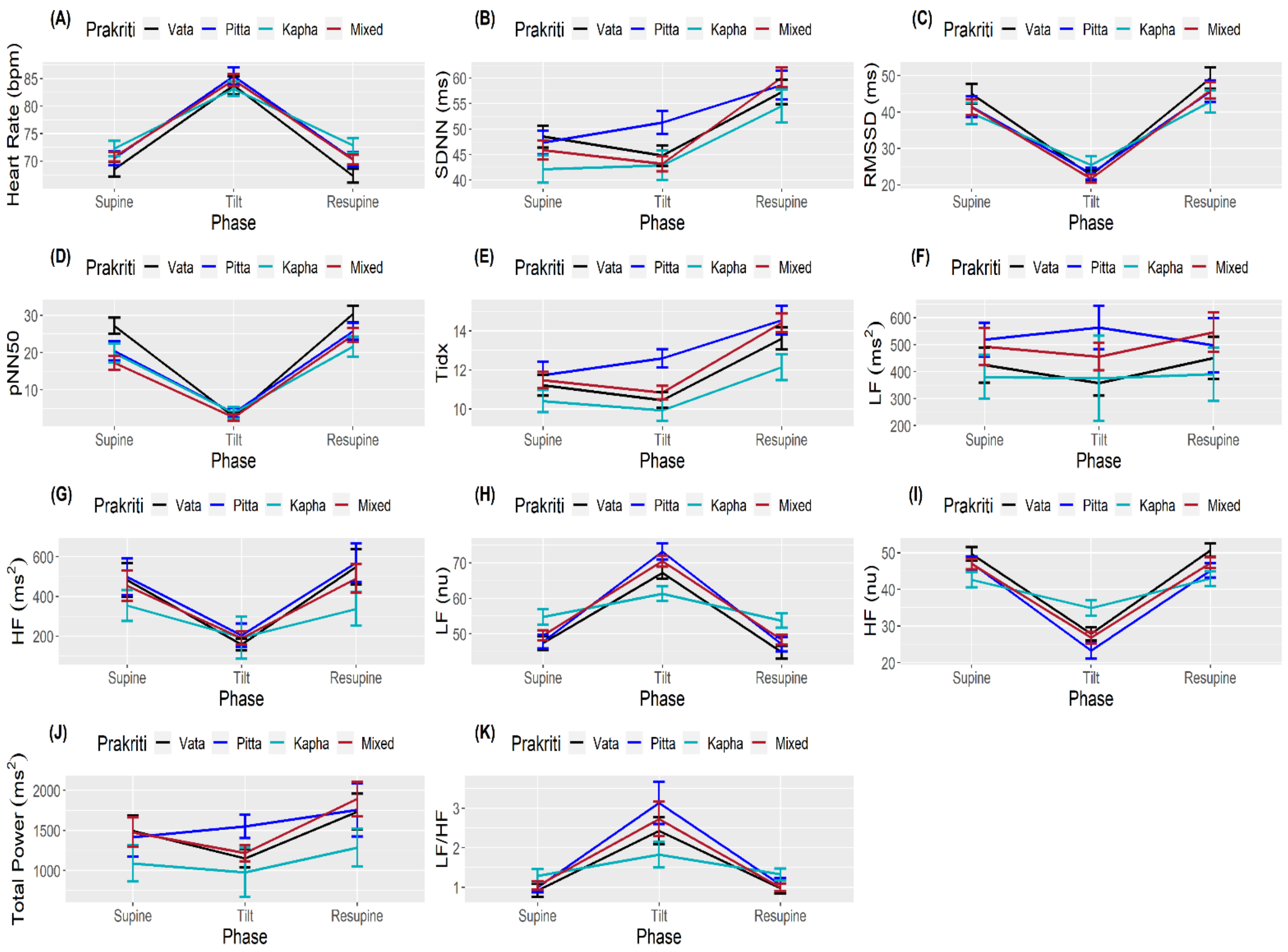

Figure S3: Line plots showing trends in Time and Frequency domain parameters of HRV among *Prakriti* groups at different phases of HUT test

Abbreviations: bpm, beats per minute; HF, high frequency; LF, low frequency; LF/HF, LF and HF ratio; nu, normalized unit; pNN50, percentage of NN50; RMSSD, root mean square of successive R-R interval differences; SDNN, standard deviation of normal to normal R-R intervals; Tidx, Triangular Index

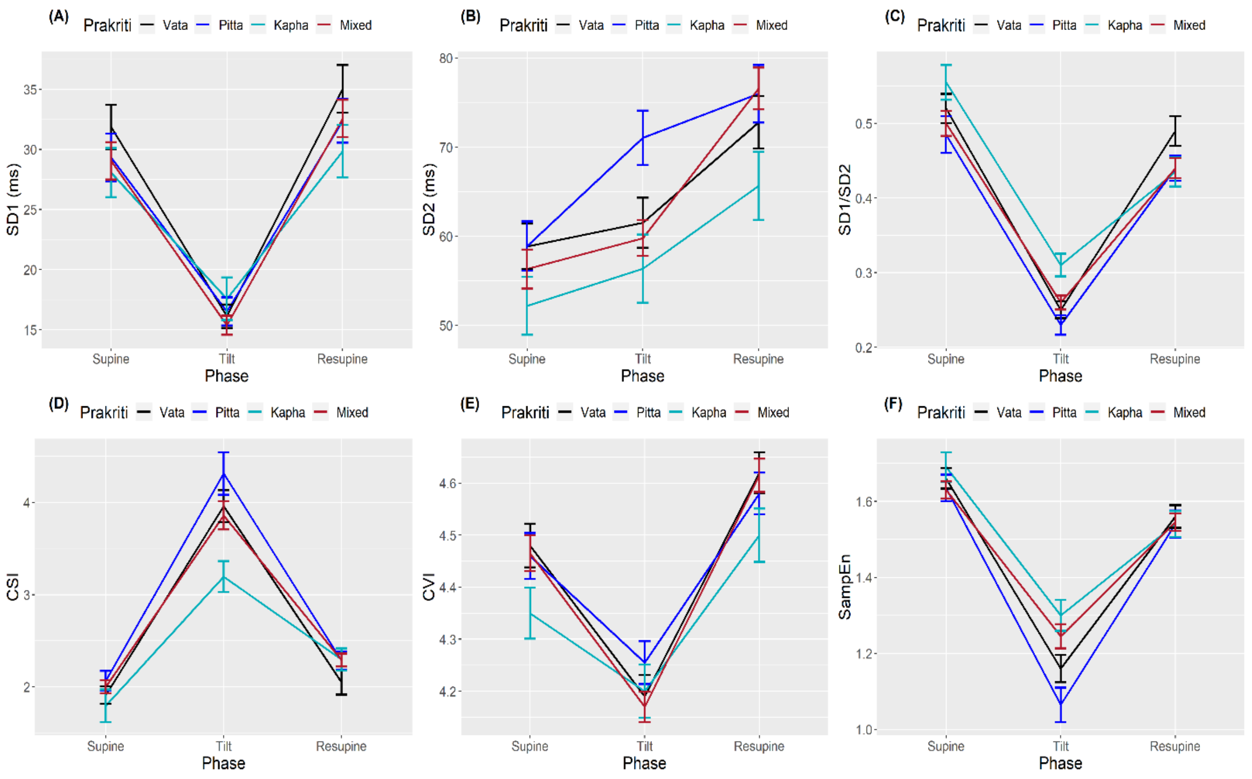

Figure S4: Line plots showing trends in non-linear parameters of HRV among *Prakriti* groups at different phases of HUT test

Abbreviations: CSI, Cardiac Sympathetic Index; CVI, Cardiac Vagal Index; SD1, standard deviation of instantaneous beat-to-beat variability; SD2, standard deviation of long-term beat to-beat variability; SampEn, Sample Entropy;

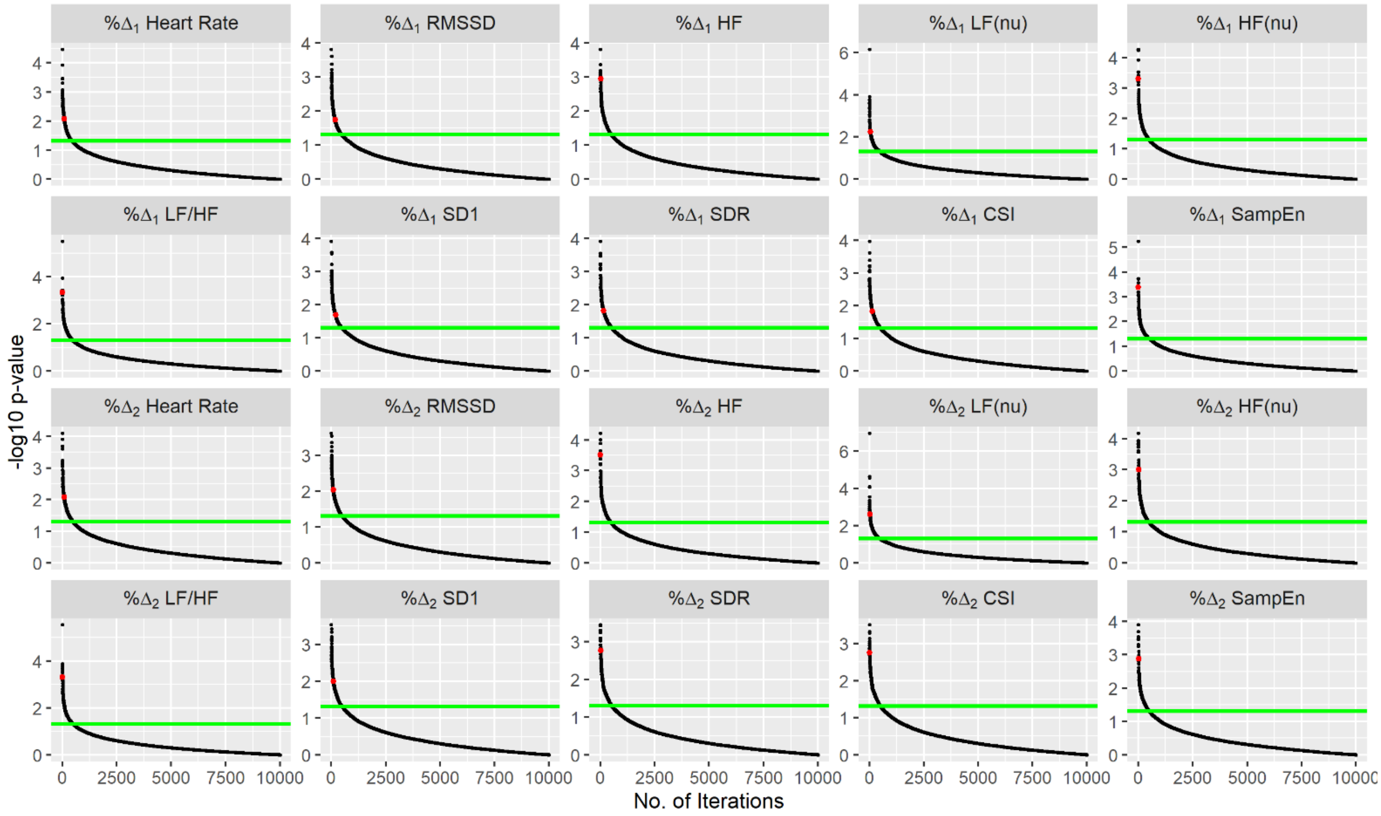

Figure S5: Validation of HRV differences amongst four *Prakriti* groups through analysis of significance performed in 10000 random iterations of same parent dataset. The dot plot showing the p-values of 10000 randomized comparisons of HRV parameters where red dot indicates original p-value of the comparison between *Vata, Pitta, Kapha* and Mixed groups and the green line indicates top 5% cut-off of permuted p-values.

Abbreviations: CSI, Cardiac Sympathetic Index; HF, high frequency; LF, low frequency; LF/HF, LF and HF ratio; nu, normalized unit; RMSSD, root mean square of successive R-R interval differences; SD1, standard deviation of instantaneous beat-to-beat variability; SD2, standard deviation of long-term beat to-beat variability; SDR: SD1/SD2; SampEn, Sample Entropy; %Δ1, relative change from supine to tilt; %Δ2, relative change from tilt to resupine

Supplementary Tables

Table S1: Heart rate variability indices (absolute values) in different *Prakriti* groups at baseline (supine) without Bonferroni Correction

Parameters	*Vata* (n=97)	*Pitta* (n=68)	*Kapha* (n=68)	Mixed (n=146)	*p*-value
**Heart Rate(bpm)**	68.51 (61.65-81.43)	70.535 (64.4-77.45)	72.265 (66.06-80.82)	70.78 (62.93-78.42)	0.446
			**Time domain**		
**SDNN(ms)**	48.52 (34.39-64.63)	47.36 (36.77-60.4)	42.14 (31.57-53.86)	45.905 (33.42-66.28)	0.237
**RMSSD(ms)**	44.99 (31.28-59.96)	41.42 (27.63-55.72)	39.63 (23.93-57.89)	41.315 (27.21-62.53)	0.568
**pNN50**	27.21 (8.35-41.03)	20.41 (5.61-37.69)	19.825 (2.95-39.69)	17.23 (4.81-45.07)	0.488
			**Geometric domain**		
**Tidx**	11.21 (9.62-15.35)	11.73 (9.61-15.25)	10.4 (8.27-12.78)	11.475 (9.11-15.07)	0.085 [a,b]
			**Frequency domain**		
**LF (ms^2)**	424.48 (243.08-807.78)	518.805 (321.56-764.08)	380.67 (193.19-697.74)	493.38 (246.15-934.98)	0.348
**HF (ms^2)**	482.2 (277.62-1061.76)	499.05 (245.38-826.18)	355.035 (125.32-729.7)	454.5 (197.84-1126.44)	0.101[a, c]
**LF (nu)**	47.37 (29.91-59.66)	47.765 (40.58-59.96)	54.725 (42.11-66.27)	49.56 (38-63.2)	0.146 [a]
**HF (nu)**	49.64 (38.34-64.52)	47.145 (36.32-56.61)	42.615 (31.09-54.57)	46.91 (33.63-57.87)	0.105 [a]
**Total Power (ms^2)**	1495.8 (818.44-2868.4)	1417.51 (968.37-3117.31)	1088.895 (670.31-2136.67)	1476.91 (711.79-2833.51)	0.168 [b]

**LF/HF**	0.92 (0.47-1.49)	1 (0.7-1.63)	1.29 (0.75-2.05)	1.04 (0.7-1.85)	0.106 [a]
			**Non-linear analysis**		
**SD1 (ms)**	31.86 (22.14-42.44)	29.33 (19.83-39.47)	28.07 (16.85-40.93)	29.045 (19.29-44.87)	0.532
**SD2 (ms)**	58.86 (40.83-80.14)	58.895 (48.03-71.64)	52.195 (41.48-64.36)	56.325 (42.19-79.3)	0.127 [a,b,c]
**SDR**	0.52 (0.46-0.63)	0.485 (0.38-0.65)	0.555 (0.42-0.66)	0.5 (0.4-0.63)	0.297
**CSI**	1.91 (1.58-2.18)	2.065 (1.54-2.63)	1.795 (1.51-2.4)	2 (1.59-2.48)	0.307
**CVI**	4.48 (4.17-4.69)	4.46 (4.16-4.63)	4.35 (4.05-4.6)	4.465 (4.12-4.77)	0.302
**SampEn**	1.66 (1.48-1.82)	1.635 (1.47-1.84)	1.69 (1.46-1.85)	1.63 (1.45-1.78)	0.733

Note: Values are expressed as median (interquartile range).

Abbreviations: BPM, beats per minutes; CSI, Cardiac Sympathetic Index; CVI, Cardiac Vagal Index; HF, high frequency; LF, low frequency; LF/HF, LF and HF ratio; ms, millisecond; nu, normalized unit; pNN50, percentage of NN50; RMSSD, root mean square of successive R-R interval differences; SampEn, Sample Entropy; SD1, standard deviation of instantaneous beat-to-beat variability; SD2, standard deviation of long-term beat to-beat variability; SDR, SD1/SD2 ratio; SDNN, standard deviation of normal to normal R-R intervals; Tidx, Triangular Index; a, *Vata* compared to *Kapha*; b, *Kapha* compared to *Pitta*; c, *Kapha* compared to Mixed; * $p < 0.05$; ** $p < 0.01$; *** $p < 0.001$; **** $p < 0.0001$; *∋ {a,b,c}.

Table S2: The Heart rate variability indices (absolute values) among different *Prakriti* groups in Tilt Phase

Parameters	*Vata* (n=97)	*Pitta* (n=68)	*Kapha* (n=68)	Mixed (n=146)	*p*-value
**Heart Rate**	83.79 (73.85-98.15)	85.47 (77.69-93.75)	83.17 (76.94-91.39)	84.735 (76.2525-92.665)	0.767
			**Time domain**		
**SDNN**	44.79 (34.62-57.01)	51.28 (40.78-65.45)	42.92 (31.44-52.52)	43.175 (33.7225-60.8425)	0.023 [b]
**RMSSD**	22.71 (15.61-30.02)	23.12 (16.52-33.39)	25.43 (15.76-35.65)	21.735 (16.195-30.9225)	0.633

**pNN50**	2.75 (0.88-7.82)	3.8 (0.9-9.68)	3.76 (0.65-14.65)	2.585 (0.7675-8.1625)	0.772
**Geometric domain**					
**Tidx**	10.45 (8.15-13.2768)	12.59 (9.53-14.31)	9.94 (7.43-12.58)	10.8455 (8.252225-13.31)	0.017 [b]
**Frequency domain**					
**LF**	358.26 (202.87-760.39)	564.16 (348.75-965.03)	375.73 (203.89-747.95)	455.73 (259.085-932.1325)	0.042
**HF**	157.78 (79.41-359.96)	205.39 (92.49-332.58)	192.9 (81.14-513.67)	191.085 (104.74-337.41)	0.605
**LF(nu)**	67.22 (55.93-79.45)	73.25 (56.17-82.38)	61.29 (50.47-73.24)	70.515 (54.0375-79.7325)	0.110
**HF(nu)**	27.87 (15.36-40.92)	23.3 (13.3-36.76)	34.92 (24.27-45.76)	26.825 (16.77-42.5)	0.022 [b]
**Total Power**	1151.87 (633.52-1846.24)	1552.32 (851.52-2318.43)	978.02 (601.21-1680.69)	1217.01 (694.47-2257.07)	0.027 [b]
**LF/HF**	2.43 (1.42-4.62)	3.14 (1.62-5.92)	1.83 (1.09-3.04)	2.725 (1.265-4.71)	0.034 [b]
**Non-linear analysis**					
**SD1**	16.11 (11.06-21.4)	16.51 (11.72-22.97)	17.56 (11.16-25.27)	15.37 (11.55-21.92)	0.691
**SD2**	61.51 (47.67-77.63)	71.05 (54.49-89.02)	56.36 (42.87-72.33)	59.79 (45.3775-83.1125)	0.016 [b]
**SDR**	0.25 (0.21-0.32)	0.23 (0.19-0.3)	0.31 (0.24-0.39)	0.26 (0.21-0.37)	0.001 [a, bb]
**CSI**	3.96 (3.1-4.79)	4.32 (3.3-5.37)	3.2 (2.59-4.12)	3.86 (2.7125-4.7075)	0.001 [a, b]
**CVI**	4.19 (3.92-4.42)	4.26 (4.08-4.47)	4.2 (3.92-4.41)	4.17 (3.9725-4.4375)	0.449
**SampEn**	1.16 (0.86-1.4)	1.07 (0.76-1.28)	1.3 (1.06-1.53)	1.245 (1.0125-1.4875)	0.001 [bb, d]

Note: Values are expressed as median (interquartile range).

Abbreviations: BPM, beats per minutes; CSI, Cardiac Sympathetic Index; CVI, Cardiac Vagal Index; HF, high frequency; LF, low frequency; LF/HF, LF and HF ratio; ms, millisecond; nu, normalized unit; pNN50, percentage of NN50; RMSSD, root mean square of successive R-R interval differences; SampEn, Sample Entropy; SD1, standard deviation of instantaneous beat-to-beat variability; SD2, standard deviation of long-term beat to-beat variability; SDR, SD1/SD2 ratio; SDNN, standard deviation of normal to normal R-R intervals; Tidx, Triangular Index; a, *Vata* compared to *Kapha*; b, *Kapha* compared to *Pitta*; c, *Kapha* compared to Mixed; d, *Pitta* compared to Mixed; * $p < 0.05$; ** $p < 0.01$; *** $p < 0.001$; **** $p < 0.0001$; *∋ {a,b,c,d}.

Table S3: The Heart rate variability indices (absolute values) among different *Prakriti* groups in Resupine Phase

Parameters	*Vata* (n=97)	*Pitta* (n=68)	*Kapha* (n=68)	Mixed (n=146)	*p*-value
**Heart Rate**	67.33 (61.14-78.18)	70.32 (64.14-75.11)	72.85 (65.62-79.28)	70.27 (61.93-76.99)	0.250
			**Time domain**		
**SDNN**	57.25 (46.77-76.36)	58.65 (44.1-74.67)	54.53 (35.81-69.01)	60.15 (42.48-76.2)	0.329
**RMSSD**	49.34 (34.63-63.35)	45.7 (34.45-59.6)	42.89 (26.17-59.91)	45.99 (30.09-66.59)	0.296
**pNN50**	30.4 (13.79-47.23)	25.71 (12.8-41.71)	21.61 (3.79-43.63)	24.72 (7.13-46.92)	0.174
			**Geometric domain**		
**Tidx**	13.61 (10.9013-17)	14.56 (11.07-18.73)	12.15 (8.97-15.8)	14.42 (10.42-17.78)	0.034 [b]
			**Frequency domain**		
**LF**	451.16 (265.62-886.54)	498.16 (335.49-795.54)	390.76 (184.38-757.22)	546.6 (263.17-1005.13)	0.286
**HF**	549.81 (298.2-1092.12)	570.08 (257.67-892.77)	337.94 (150.26-738.08)	490.67 (250.68-1177.73)	0.038 [a]
**LF(nu)**	44.78 (32.94-57.23)	47.02 (35.84-58.96)	53.69 (42.9-64.27)	48.26 (38.11-61.51)	0.077
**HF(nu)**	50.68 (34.86-63.6)	45.14 (34.31-56.33)	42.94 (29.79-52.87)	47.23 (33.01-58.31)	0.049 [a]
**Total Power**	1734.63 (1033.64-2835.42)	1755.01 (1109.55-3135.75)	1284.21 (607.53-2897.79)	1894.27 (892.53-2947.59)	0.141

**LF/HF**	0.97 (0.55-1.68)	1.07 (0.65-1.73)	1.33 (0.87-2.06)	0.99 (0.67-1.89)	0.053 [a]
			**Non-linear analysis**		
**SD1**	35.04 (24.52-45.68)	32.37 (24.41-42.27)	29.86 (18.54-42.43)	32.57 (21.66-47.14)	0.281
**SD2**	72.78 (57.66-91.68)	75.99 (57.4-94.11)	65.65 (46.68-88.18)	76.58 (55.03-99.37)	0.248
**SDR**	0.49 (0.37-0.6)	0.44 (0.34-0.53)	0.44 (0.36-0.53)	0.44 (0.34-0.56)	0.218
**CSI**	2.05 (1.66-2.69)	2.29 (1.91-2.92)	2.3 (1.89-2.78)	2.29 (1.79-2.95)	0.219
**CVI**	4.62 (4.35-4.85)	4.58 (4.38-4.82)	4.5 (4.11-4.72)	4.62 (4.29-4.83)	0.289
**SampEn**	1.56 (1.36-1.75)	1.54 (1.36-1.71)	1.54 (1.37-1.76)	1.55 (1.35-1.7)	0.895

Note: Values are expressed as median (interquartile range).

Abbreviations: BPM, beats per minutes; CSI, Cardiac Sympathetic Index; CVI, Cardiac Vagal Index; HF, high frequency; LF, low frequency; LF/HF, LF and HF ratio; ms, millisecond; nu, normalized unit; pNN50, percentage of NN50; RMSSD, root mean square of successive R-R interval differences; SampEn, Sample Entropy; SD1, standard deviation of instantaneous beat-to-beat variability; SD2, standard deviation of long-term beat to-beat variability; SDR, SD1/SD2 ratio; SDNN, standard deviation of normal to normal R-R intervals; Tidx, Triangular Index; a, *Vata* compared to *Kapha*; b, *Kapha* compared to *Pitta*; c, *Kapha* compared to Mixed; d, *Pitta* compared to Mixed; * $p < 0.05$; ** $p < 0.01$; *** $p < 0.001$; **** $p < 0.0001$

Table S4: The Heart rate variability indices among Mixed *Prakriti* groups at baseline (rest in supine position)

		**Baseline**		
**Parameters**	**Kapha-Pitta (n=51)**	**Vata-Kapha (n=13)**	**Vata-Pitta (n=82)**	**P-value**
**Heart Rate(bpm)**	70.87(62.66-77.92)	70.36(65.22-72.08)	71.93(62.61-79.05)	0.607
		**Time domain**		
**SDNN (ms)**	43.41(30.44-65.91)	41.23(32.13-70.6)	49.91(36.58-64.87)	0.459
**RMSSD (ms)**	36.19(26.22-57.04)	33.7(22.78-68.71)	47.71(29.52-65.64)	0.405

**pNN50**	16.02(5.1-32.32)	11.7(2.19-33.95)	25.98(8.26-46.77)	0.258
**Geometric domain**				
**Tidx**	10.84(8.47-14.24)	10.64(7.41-11.06)	12.91(9.68-15.93)	0.047
**Frequency domain**				
**LF (ms^2)**	473.25(246.02-892)	389.83(271.2-888.28)	523.24(246.15-967.84)	0.888
**HF (ms^2)**	411.27(189.98-982.74)	323.86(192.64-719.21)	578.66(235.41-1180.72)	0.308
**LF (nu)**	49.8(44.74-63.05)	59.81(41.2-67.54)	47.77(35.32-61.66)	0.135
**HF (nu)**	46(33.53-55.05)	40.19(32.46-58.8)	48.96(34.25-59.15)	0.579
**Total Power (ms^2)**	1334.67(678.51-2423.17)	1276.93(586.66-3528.73)	1666.68(781.93-3072.69)	0.292
**LF/HF**	1.17(0.82-1.83)	1.49(0.7-2.08)	0.95(0.62-1.76)	0.310
**Non-linear analysis**				
**SD1 (ms)**	25.63(18.58-40.39)	23.86(16.13-48.66)	33.79(20.91-46.49)	0.405
**SD2 (ms)**	55.2(38.46-82.95)	53.61(42.55-81.12)	58.27(47.14-77.54)	0.603
**SDR**	0.5(0.42-0.61)	0.52(0.39-0.61)	0.5(0.39-0.66)	0.930
**CSI**	2(1.65-2.37)	1.93(1.63-2.58)	2.01(1.52-2.58)	0.927
**CVI**	4.37(4.08-4.75)	4.34(4.04-4.72)	4.51(4.15-4.77)	0.525
**SampEn**	1.71(1.52-1.84)	1.72(1.48-1.87)	1.59(1.43-1.72)	0.049

Note: Values are expressed as median (interquartile range).

Abbreviations: BPM, beats per minutes; CSI, Cardiac Sympathetic Index; CVI, Cardiac Vagal Index; HF, high frequency; LF, low frequency; LF/HF, LF and HF ratio; ms, millisecond; nu, normalized unit; pNN50, percentage of NN50; RMSSD, root mean square of successive R-R interval differences; SampEn, Sample Entropy; SD1, standard deviation of instantaneous beat-to-beat variability; SD2, standard deviation of long-term beat to-beat variability; SDR, SD1/SD2 ratio; SDNN, standard deviation of normal to normal R-R intervals; Tidx, Triangular Index;

Table S5: The relative change (*%Δ₁*) of Heart Rate Variability indices among Mixed Prakriti groups in response to orthostatic stress than supine to tilt position

Parameters	Kapha-Pitta (n=51)	Vata-Kapha (n=13)	Vata-Pitta (n=82)	P-value
***$\%\Delta_1$* Heart Rate**	12.99(6.61-19.27)	14.57(11.26-17.6)	17.28(10.19-30.87)	0.035[a]
**Time domain**				
***$\%\Delta_1$* SDNN**	3.27(-21.88-27.3)	9.51(-5.78-14.45)	-7.6(-30.71-24.14)	0.415
***$\%\Delta_1$* RMSSD**	-35.38(-49.69--22.46)	-21.83(-48.9--17.56)	-47.7(-67.62--26.05)	0.041
***$\%\Delta_1$* pNN50**	-77.78(-90.79--46.43)	-32.74(-80.2--19.44)	-83.51(-97.89--60.65)	0.012[b]
**Geometric domain**				
***$\%\Delta_1$* Tidx**	2.64(-16.78-15.96)	7.4(-16.47-20.72)	-14.46(-32.49-16.44)	0.249
**Frequency domain**				
***$\%\Delta_1$* LF**	5.15(-36.31-53.33)	31.07(-28.36-71.41)	-8.25(-44.34-74.05)	0.767
***$\%\Delta_1$* HF**	-44.36(-68.74--6.17)	-26.75(-67.29-10.07)	-63.5(-84.88--30.31)	0.019[a]
***$\%\Delta_1$* LF(nu)**	17.05(-2.13-47.76)	14.04(4.66-29.62)	44.71(11.01-86.6)	0.025[a]
***$\%\Delta_1$* HF(nu)**	-22.33(-50.07-1.84)	-37.71(-55.91--16.8)	-47.58(-67.24--16.28)	0.045[a]
***$\%\Delta_1$* Total Power**	-3.19(-37.35-31.6)	13.69(-27.8-25.62)	-23.92(-47.8-29.18)	0.502
***$\%\Delta_1$* LF/HF**	61.81(-4.97-263.76)	93.51(25.83-212.69)	201.86(33.63-456.19)	0.042[a]
**Non-linear analysis**				
***$\%\Delta_1$* SD1**	-35.33(-49.67--22.4)	-21.82(-48.87--17.42)	-46.93(-67.6--25.93)	0.045
***$\%\Delta_1$* SD2**	8.14(-13.14-39.16)	11.36(-4.08-20.19)	1.71(-19.1-36.24)	0.642
***$\%\Delta_1$* SDR**	-40(-53.58--27.82)	-35.9(-49.18--25.93)	-50.49(-59.14--29.23)	0.089

**%Δ_1 CSI**	64.86(40.32-114.59)	53.49(34.97-100)	99.9(42.66-145.01)	0.081
**%Δ_1 CVI**	-3.54(-8.08-0.83)	-0.99(-3.71--0.27)	-5.32(-12.33--0.47)	0.083
**%Δ_1 SampEn**	-18.71(-31--4.29)	-17.78(-34.43--6.81)	-19.82(-41.2--7.34)	0.653

Note: Values are expressed as median (interquartile range).

Abbreviations: BPM, beats per minutes; CSI, Cardiac Sympathetic Index; CVI, Cardiac Vagal Index; HF, high frequency; LF, low frequency; LF/HF, LF and HF ratio; ms, millisecond; nu, normalized unit; pNN50, percentage of NN50; RMSSD, root mean square of successive R-R interval differences; SampEn, Sample Entropy; SD1, standard deviation of instantaneous beat-to-beat variability; SD2, standard deviation of long-term beat to-beat variability; SDR, SD1/SD2 ratio; SDNN, standard deviation of normal to normal R-R intervals; Tidx, Triangular Index; a, *Kapha-Pitta* compared to *Vata-Pitta*; b, *Vata-Pitta* compared to *Vata-Kapha*; * $p < 0.05$; ** $p < 0.01$; *** $p < 0.001$; **** $p < 0.0001$

Table S6: The relative change (%Δ_2) of Heart Rate Variability indices among Mixed Prakriti groups during recovery response than tilt to resupine position

Parameters	Kapha-Pitta (n=51)	Vata-Kapha (n=13)	Vata-Pitta (n=82)	P-value
**%Δ_2 Heart Rate**	-12.97(-18.16--6.39)	-10.2(-12.24--9.74)	-16.49(-24.61--11.71)	0.003[a,b]
		**Time domain**		
**%Δ_2 SDNN**	18.33(-7.43-59.28)	34.54(12.64-54)	34.94(6.54-76.78)	0.195
**%Δ_2 RMSSD**	62.09(19.19-125.7)	60.97(44.22-109.9)	124.83(57.33-198.95)	0.005[aa]
**%Δ_2 pNN50**	340.1(64.68-1188.21)	386.18(90.58-634.15)	436.9(181.04-1397.62)	0.431
		**Geometric domain**		
**%Δ_2 Tidx**	13.14(-4.93-44.81)	23.26(13-40.19)	35.3(10.74-67.82)	0.038[a]
		**Frequency domain**		
**%Δ_2 LF**	2.98(-38.35-103.03)	37.04(-46.15-58.25)	13.66(-33.71-90.17)	0.961
**%Δ_2 HF**	83.42(15.8-265.58)	81.46(20.99-249.12)	237.41(55.3-486.59)	0.011[a]
**%Δ_2 LF(nu)**	-18.38(-34.09--0.86)	-19.82(-36.93-2.67)	-30.82(-47.95--7.33)	0.042

**%Δ_2 HF(nu)**	37.77(-0.12-93.38)	75.74(-6.46-113.73)	81.99(10.79-248.47)	0.037[a]
**%Δ_2 Total Power**	23.64(-15.24-128.85)	69.41(-13.99-107.13)	37.45(-4.76-96.07)	0.679
**%Δ_2 LF/HF**	-41.06(-66.54-0.4)	-57.84(-69.33-10.96)	-65.42(-85.15--16)	0.022[a]
		**Non-linear analysis**		
**%Δ_2 SD1**	59.86(19.09-125.35)	60.86(44.18-109.81)	123.4(56.83-199.36)	0.005[aa]
**%Δ_2 SD2**	9.2(-11.25-53.93)	29.87(8.75-50.08)	24.42(-3.27-68.19)	0.287
**%Δ_2 SDR**	37.84(15.21-88)	32(10-55)	76.44(27.14-134.58)	0.040
**%Δ_2 CSI**	-27.49(-45.96--12.47)	-23.48(-35.02--7.07)	-43.01(-56.48--21.97)	0.035
**%Δ_2 CVI**	6.03(1.41-12.18)	7.73(4.62-11.97)	10.27(5.59-16.16)	0.020[a]
**%Δ_2 SampEn**	14.06(1.89-40.22)	6.45(-6.8-22.64)	21.2(-2.9-70.37)	0.317

Note: Values are expressed as median (interquartile range).

Abbreviations: BPM, beats per minutes; CSI, Cardiac Sympathetic Index; CVI, Cardiac Vagal Index; HF, high frequency; LF, low frequency; LF/HF, LF and HF ratio; ms, millisecond; nu, normalized unit; pNN50, percentage of NN50; RMSSD, root mean square of successive R-R interval differences; SampEn, Sample Entropy; SD1, standard deviation of instantaneous beat-to-beat variability; SD2, standard deviation of long-term beat to-beat variability; SDR, SD1/SD2 ratio; SDNN, standard deviation of normal to normal R-R intervals; Tidx, Triangular Index; a, *Kapha-Pitta* compared to *Vata-Pitta*; b, *Vata-Pitta* compared to *Vata-Kapha*; * $p < 0.05$; ** $p < 0.01$; *** $p < 0.001$; **** $p < 0.0001$
